# Revealing the Differences in Microbial Community and Quality of High-Temperature Daqu in the Southern Sichuan–Northern Guizhou Region

**DOI:** 10.3390/foods14040570

**Published:** 2025-02-08

**Authors:** Cheng Yan, Zhangjun Huang, Rongkun Tu, Liqiang Zhang, Chongde Wu, Songtao Wang, Ping Huang, Yunhang Zeng, Bi Shi

**Affiliations:** 1 College of Biomass Science and Engineering, Sichuan University, Chengdu 610065, China; yancheng200625@outlook.com (C.Y.); cdwu@scu.edu.cn (C.W.); 2024323080024@stu.scu.edu.cn (P.H.); shibi@scu.edu.cn (B.S.); 2 National Engineering Research Center of Solid-State Brewing, Luzhou 646000, China; huangzj0331@163.com (Z.H.); turk@lzlj.com (R.T.); zhanglq@lzlj.com (L.Z.); wangst@lzlj.com (S.W.); 3 Luzhou Laojiao Co., Ltd., Luzhou 646000, China

**Keywords:** Baijiu, high-temperature Daqu, metagenomics, microbial function, volatile compound, microbial interaction, functional enzyme

## Abstract

High-temperature Daqu is crucial to Jiang-flavor Baijiu production in the Southern Sichuan–Northern Guizhou region of China. However, the complex interplay among microorganisms, enzymes, and metabolites in the Daqu from this region requires further investigation. This study compared four high-temperature Daqu samples from this region, analyzing their physicochemical properties, enzyme activities, volatile compounds, and microbial community composition and function, and exploring the influence of microorganisms on the saccharification and aroma-formation function of Daqu in combination with correlation analysis. The microbial communities in the Daqu samples exhibited functional redundancy, with *Desmospora* sp. 8437 being consistently dominant (3.6–7.3%). Members of the family *Bacillaceae* were the principal factors contributing to the differences in starch degradation capacity, protein degradation capacity, and pyrazine content among the Daqu samples, mainly through the amylases and proteases they produce. *Kroppenstedtia* spp. were principal factors causing the differences in aldehyde and ketone contents, primarily via the lipid degradation enzymes they synthesize. Overall, the bacterial community composition of Daqu greatly influenced its characteristics. This study provided a theoretical basis for understanding the diversity of high-temperature Daqu in the Southern Sichuan–Northern Guizhou region.

## 1. Introduction

Jiang-flavor Baijiu, a major segment of the Chinese Baijiu market [1], possesses a distinctive aroma profile derived from specific volatile compounds produced during fermentation. In the traditional solid-state fermentation of Jiang-flavor Baijiu, high-temperature Daqu plays an essential role [2]. High-temperature Daqu is a product obtained from wheat through spontaneous inoculation, fermentation, and complex biochemical changes at temperatures reaching from 60 °C to 75 °C [3]. Following milling and mixing, mature high-temperature Daqu is added to the fermentation system. The microorganisms, which are a core component of the high-temperature Daqu used in Daqu fermentation and Baijiu brewing [4], contribute to differences in physicochemical properties, enzyme activities, and volatile metabolites among various types of Daqu [5]. These differences are intrinsically linked to Daqu quality and influence the final product, Jiang-flavor Baijiu [6]. Microorganisms play crucial roles in saccharification and aroma production. For example, *Kroppenstedtia*, *Bacillus*, and *Aspergillus* metabolize amylases, providing enzymes for saccharification, accelerating starch hydrolysis, and promoting alcohol fermentation [7]. In terms of aroma production, both *Kroppenstedtia* and *Bacillus* influence pyrazine formation [8,9], while *Brevibacterium linens* may contribute to the synthesis of sulfur-containing compounds [6]. However, the composition of Daqu’s microbial community is complex, and the interaction mechanisms between microorganisms are not fully understood. This complexity makes precise regulation of the Daqu microbial community challenging. Therefore, in-depth exploration of the structure and function of the high-temperature Daqu microbial community remains important in microbiology and brewing engineering, holding significant potential for optimizing Daqu fermentation and improving Baijiu quality.

The Southern Sichuan–Northern Guizhou region is the core production area for Jiang-flavor Baijiu with diverse aroma profiles. As a key fermentation starter for Jiang-flavor Baijiu, high-temperature Daqu contributes significantly to this diversity through its varied microbial communities, enzymes, and metabolites [8,10]. Therefore, the research on high-temperature Daqu in this region has always been a focus of traditional fermentation research. Over the past years, amplicon sequencing has been widely used to study the microbial communities of high-temperature Daqu. Using this method, Zuo et al. [11] examined the effect of a molding method on genus-level microbial communities and found a higher abundance of *Bacillus* in machine-made Daqu than in handmade Daqu. Shi et al. [12] investigated the relationship between genus-level microbial communities and volatile compounds in high-temperature Daqu of different colors. However, a systematic comparison of microbial communities, enzymes, and volatile metabolites across various high-temperature Daqu from this region remains lacking.

With the development of high-throughput sequencing technologies, metagenomics has been extensively applied in the research of Daqu [13,14] for species level taxonomic resolution and functional prediction to enhance the understanding of microbial community structure and function [15]. Therefore, this study employed shotgun metagenomics and headspace-solid phase microextraction gas chromatography–mass spectrometry (HS-SPME-GC-MS)-based metabolomics to characterize the microbial composition, functional profiles, and volatile organic compound profiles of four representative high-temperature Daqu samples from the Southern Sichuan–Northern Guizhou region. Bioinformatics analyses at the microbial and enzymatic levels elucidated the underlying causes of variations in the main characteristics of high-temperature Daqu. The findings further clarify the microbial community structure and enzyme systems of the high-temperature Daqu from in this region and provide data support for its style regulation.

## 2. Materials and Methods

### 2.1. Sample Collection

As shown in Figure S1, four high-temperature Daqu samples were collected from the area where the Yangtze River meets the Tuojiang River and from the middle reaches of the Chishui River in the Southern Sichuan and Northern Guizhou regions. The high-temperature Daqu samples from Southern Sichuan were named SJ and ZS (29°02′ N, 105°44′ E and 29°87′ N, 105°56′ E), and those from Northern Guizhou were labeled MX and ZJ (27°82′ N, 106°32′ E and 27°92′ N, 106°36′ E). All samples were mature high-temperature Daqu prepared through traditional fermentation. Specifically, wheat was selected, screened, and cleaned of impurities. The wheat was then crushed, moistened to a water content of 35–45%, and mixed with 3–5% mother Daqu powder. This mixture was formed into Daqu cakes of specific shapes and sizes and placed in a Daqu-making room for fermentation. Temperature and humidity were regulated by regularly turning the Daqu. The fermentation temperature reached a maximum of over 60 °C, and the entire cycle lasted 30–45 days. Following fermentation, the Daqu was stored for 3–6 months to reach maturity, after which it was used as a saccharification and fermentation agent in Jiang-flavor Baijiu production [8]. The four high-temperature Daqu samples used in this study were all produced in mid-August to minimize seasonal effects. A nine-point stratified sampling method was used to reduce the variations in the microbial community within Daqu, resulting from their location in the Daqu-making room. This method ensured a comprehensive representation of the microbial community from various locations within each of the four mature Daqu samples. Nine samples were collected per Daqu type, which were then pooled, homogenized, and divided into three parts to obtain four representative samples for subsequent analyses. One portion of each sample was stored at 4 °C for the analysis of physicochemical indicators and enzyme activities. Another portion was stored at −20 °C for volatile compound analysis to prevent degradation, and the final portion was stored at −80 °C for DNA extraction and microbial community analysis to ensure the integrity of the genetic material.

### 2.2. Determination of Physicochemical Properties and Enzymatic Activities

Moisture content, acidity, starch content, reducing sugar content, amino acid nitrogen content, liquefaction power, saccharification power, fermentation power, and esterification power were determined using standard techniques [16]. Acidity was measured via potentiometric titration using acid-base neutralization and expressed as millimoles of 0.1 mol/L sodium hydroxide standard solution consumed per 10 g of dried Daqu. Amino acid nitrogen content was determined based on the amphoteric nature of amino acids. Following the addition of formaldehyde, the carboxyl group was titrated with sodium hydroxide standard solution, and the nitrogen content was calculated based on the consumption of sodium hydroxide, expressed in grams per kilogram (g/kg). Liquefaction power was defined as the grams of starch liquefied by 1 g of dried Daqu in 1 h at 35 °C and pH 4.6, indicating the ability of Daqu to hydrolyze starch into shorter chains or small molecular sugars (unit: g/g·h). Saccharification power was defined as the milligrams of glucose generated by 1 g of dried Daqu in 1 h from the conversion of soluble starch at 35 °C and pH 4.6, reflecting Daqu’s saccharifying amylase activity in hydrolyzing starch into monosaccharides (unit: mg/g·h). Fermentation power was defined as the grams of carbon dioxide produced by 0.5 g of Daqu from fermentable carbohydrates within 72 h at 30 °C, reflecting the ability of Daqu’s microorganisms to ferment sugars into alcohol, carbon dioxide, and other metabolites (unit: g/0.5 g·72 h). Esterification power was defined as the milligrams of ethyl hexanoate synthesized by 50 g of Daqu catalyzing hexanoic acid and ethanol for 7 days at 35 °C, indicating Daqu’s esterifying enzyme activity in synthesizing ester compounds from organic acids and alcohols (unit: mg/50 g·7 d).

The activity of high-temperature resistant amylase (HTR-amylase) was determined using the DNS method and was defined as the number of milligrams of reducing sugar converted from soluble starch per hour at 35 °C and pH 4.6 after 1 g of dried Daqu was pretreated at 70 °C for 15 min (mg/g·h) [16,17,18]. The activity of cellulase was determined using the CMCA-DNS method and was defined as the number of milligrams of reducing sugar hydrolyzed from sodium carboxymethyl cellulose per hour at 50 °C and pH 4.6 per gram of dried Daqu (mg/g·h) [16,19]. The activities of neutral/acidic proteases were determined using the Folin reagent method and were defined as the amount of tyrosine hydrolyzed from casein per minute at 40 °C and pH 7.2/3.0, expressed in micrograms per gram of dried Daqu (μg/g·min) [16,20]. The activity of carboxylesterase was determined using the fast blue colorimetric method and was defined as the amount of α-naphthyl ester hydrolyzed from α-naphthyl acetate per minute at 37 °C and pH 6.0, expressed in millimoles per gram of dried Daqu (mmol/g·min) [16,21].

Three independent replicates were carried out for each of the assays mentioned above to ensure the accuracy and reliability of the experimental data. Each replicate was performed under the same experimental conditions and operating procedures, and the final data were the average of the three measurements.

### 2.3. Analysis of Volatile Metabolites by HS-SPME-GC/MS

The volatile compounds in high-temperature Daqu were extracted by HS-SPME. In brief, 0.5 g of the sample (dry basis), 5 mL of pure water, 2 g of NaCl, and 10 μL of 2-octanol (0.0151 g/100 mL) were mixed in a 15 mL headspace vial and equilibrated at 60 °C for 5 min. The extraction fiber (DVB/CAR/PDMS fiber with a 50/30 μm three-phase extraction head) was then inserted into the headspace vial to extract the sample for 50 min at the same temperature.

Immediately after extraction, the extraction fiber was introduced into the injection port of the Shimadzu (Kyoto, Japan) GCMS-QP2020 NX system (thermally desorbed at 250 °C for 5 min). Separation was carried out using a DB-WAX column (30 m × 0.25 mm, 0.25 μm), with helium gas at a flow rate of 1.0 mL/min as the carrier gas. The temperature program was as follows: held at 40 °C for 5 min, then increased to 100 °C at a rate of 4 °C/min and further to 230 °C at a rate of 6 °C/min for 10 min. The mass spectra were acquired in the electron ionization mode at 70 eV, within the mass range of *m*/*z* 33–450. The temperatures of the ion source and the interface were set to 230 °C and 250 °C, respectively. Compounds were identified by comparing their mass spectra with the NIST20s spectral database. Quantification was performed using the internal standard method.

### 2.4. Metagenomic Sequencing and Data Processing

Total genomic DNA was extracted from the four Daqu samples using the Mag-Bind^®^ Soil DNA Kit (Omega Bio-tek, Norcross, GA, USA) according to the manufacturer’s instructions. The concentration and purity of extracted DNA were determined with TBS-380 (Turner Biosystems, Sunnyvale, CA, USA) and NanoDrop2000 (Thermo Fisher Scientific, Waltham, MA, USA), respectively. The quality of the DNA extract was checked on a 1% agarose gel. The extracted microbial DNA extracts were sonicated using Covaris M220 (Covaris, Woburn, MA, USA) to fragment them to an average size of 350 bp. Paired-end libraries were then constructed using NEXTFLEX Rapid DNA-Seq (Bioo Scientific, Austin, TX, USA) by ligating the adapters containing the full set of sequencing primer hybridization sites to the blunt ends of the fragments. Paired-end sequencing was carried out on the Illumina NovaSeq platform (Illumina Inc., San Diego, CA, USA) of Majorbio Bio-Pharm Technology Co., Ltd. (Shanghai, China) using the NovaSeq6000 S4 Reagent Kit v1.5 (300 cycles).

The raw data were processed to obtain quality-filtered reads for further analysis. First, Fastp (v0.20.0) was used to trim off the low-quality bases and adapter sequences in the data. BWA (v0.717) was then employed to align the remaining reads with the host genome to remove host contamination.

After the clean data were obtained, MEGAHIT v1.1.2 was used to assemble the short sequencing reads into contiguous sequences with a length greater than 300 bp. In addition, Prodigal v2.6.3 (https://github.com/hyattpd/Prodigal (accessed on 2 May 2024)) was applied to predict the open reading frames (ORFs) of the assembled contiguous sequences. CD-HIT v4.6.1 was employed to cluster the predicted gene sequences to obtain a nonredundant gene set. The abundance information of the genes in each sample was obtained from the alignment information by SOAPaligner. With the use of Diamond v2.0.13 and the Best-Hit species annotation method, the nonredundant gene set was aligned with the NR database to obtain the species and abundance information at each taxonomic level in each sample (E-value ≤ 1 × 10^−5^). Similarly, the functional profiles of nonredundant genes were obtained by annotating the KEGG, CAZy, and COG databases using the DIAMOND alignment algorithm. Pathways and enzymes associated with the saccharification and aroma-formation function of Daqu were also constructed using the KEGG database and the relevant literature [22,23,24,25].

### 2.5. Statistical Analysis and Visualization

PCA, α-diversity analysis, Kruskal–Wallis rank sum test, and LEfSe analysis were completed on the Majorbio Cloud Platform. Alpha diversity analysis was conducted using the Chao, ACE, Simpson, and Shannon indices. A higher Chao index indicates a greater number of operational taxonomic units (OTUs), reflecting a relatively high level of species richness within the sample. The ACE index, similar to the Chao index, estimates species richness. The Simpson index measures the probability that two randomly selected individuals from the sample belong to the same species, thereby reflecting species dominance. The Shannon index integrates species richness and evenness. A higher the Shannon index signifies greater community diversity with a more uniform abundance distribution. Venn diagrams were constructed to visualize the shared and unique volatile metabolites in the Daqu samples using a free online website (http://www.ehbio.com/test/venn/#/ (accessed on 2 November 2024)). The co-occurrence network between microorganisms was visualized using the R packages dplyr (v1.1.4) and stats (v4.4.1) and the Gephi program (v0.9.2). Spearman rank correlation analysis was carried out using the R packages corrplot (v0.95) and psych (v2.4.6.26) to investigate the interactions between the major microorganisms and the physicochemical indicators, enzyme activities, and volatile compounds. Bubble plots showing the abundances of key functional enzymes in each sample were generated using the R package ggplot2 (v3.5.1). Histograms and pie charts were completed by Origin 2021 (OriginLab Corporation, Northampton, MA, USA).

## 3. Results

### 3.1. Analysis of Physicochemical Properties and Enzymatic Activities

**Figure 1:**
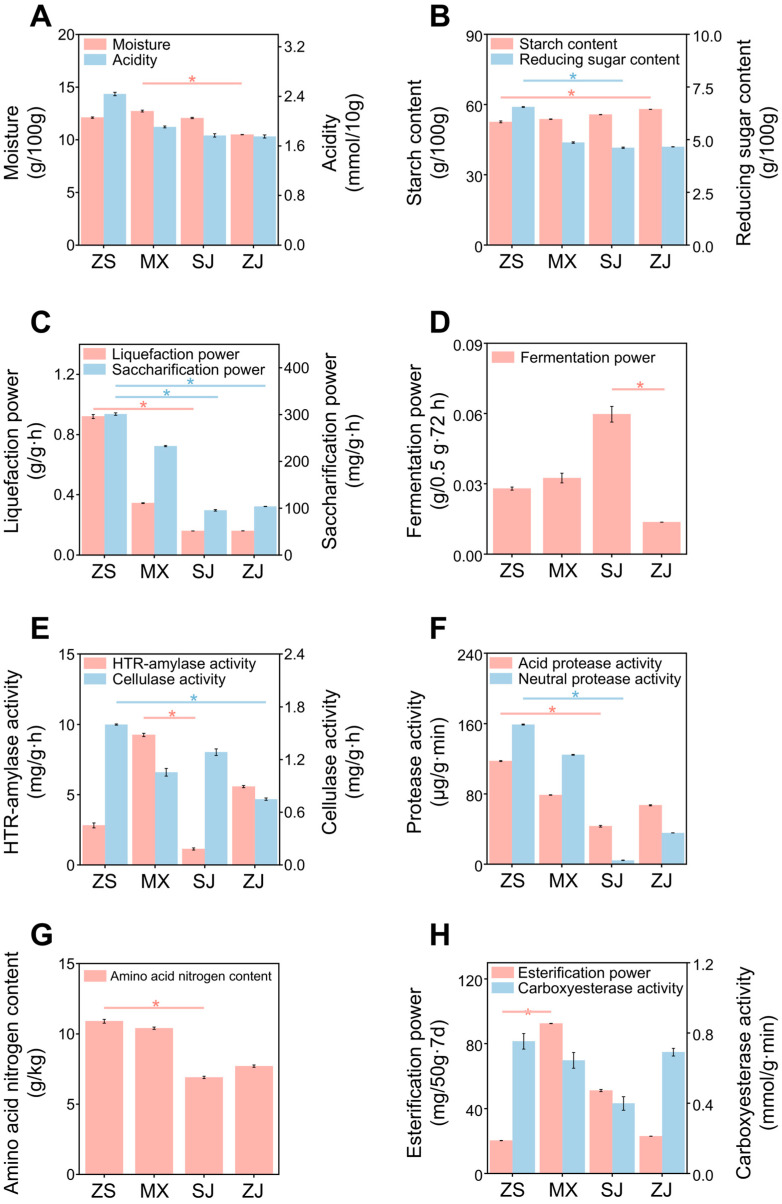
Physicochemical properties and enzymatic activities of four high-temperature Daqu samples (ZS, MX, SJ, and ZJ). (**A**) Moisture and acidity, (**B**) starch content and reducing sugar content, (**C**) liquefaction power and saccharification power, (**D**) fermentation power, (**E**) HTR-amylase activity and cellulase activity, (**F**) Neutral and acid protease activity, (**G**) amino acid nitrogen content, and (**H**) esterification power and carboxyesterase activity. Significant differences between samples for each index were analyzed using Kruskal–Wallis rank sum test. Samples with significant differences are indicated by asterisk (*) (*p* < 0.05).

Significant variations in physicochemical properties and enzyme activities were observed among the four Daqu samples (Figure 1). ZS exhibited the highest acidity (2.44 ± 0.03 mmol/10 g) and reducing sugar content (6.55 ± 0.02 g/100 g) and strong saccharification and cellulase activity (301 ± 4 mg/g·h and 0.92 ± 0.02 mg/g·h), indicating its strong starch degradation. MX showed the highest HTR-amylase activity (9.3 ± 0.1 mg/g·h). ZS and MX had higher acidic protease activity (117.6 ± 0.7 and 78.8 ± 0.3 μg/g·min), neutral protease activity (159.1 ± 0.5 and 124.7 ± 0.5 μg/g·min), and amino acid nitrogen content (10.9 ± 0.2 and 10.4 ± 0.1 mol/L) than SJ and ZJ, indicating their strong amino acid metabolism. The highest fermenting power (0.06 ± 0.00 g/0.5 g·72 h) was observed for SJ, suggesting its efficient utilization of reducing sugars that is consistent with its relatively low reducing sugar content (Figure 1D). PCA further confirmed the diversity in physicochemical properties and enzymatic activities among the four high-temperature Daqu types (Figure S2). The variances between ZS/MX and SJ/ZJ were due to their differences in liquefaction power, saccharification power, reducing sugar content, and protease activity. In particular, SJ differed from the other samples primarily due to its esterifying power, fermenting power, and cellulase activity. ZJ varied with the other samples mainly in terms of starch content and carboxylesterase activity.

### 3.2. Analysis of Volatile Compounds

**Figure 2:**
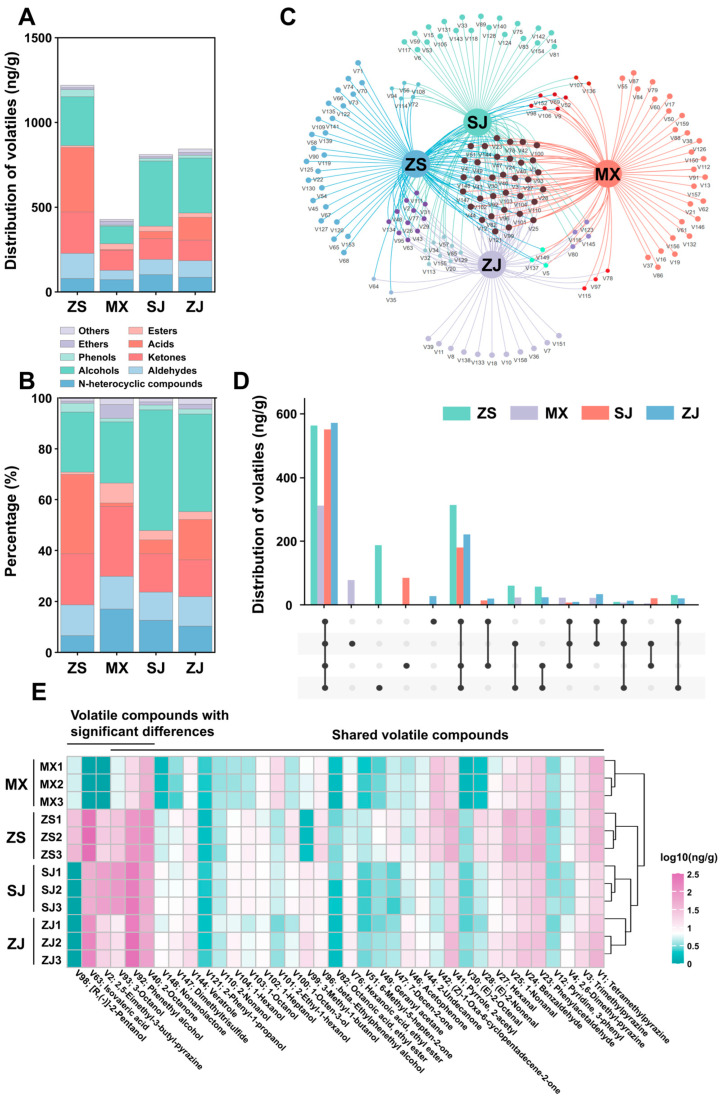
A comparison of the volatile compounds of the four high-temperature Daqu samples based on the results of HS−SPME−GC/MS. (**A**) Variations in the category and number of volatiles, (**B**) variations in the relative content of each category, (**C**) shared and unique volatile compounds presented by Venn networks, (**D**) relative abundances of shared and unique volatile compounds shown by Upset plot, and (**E**) contents of the top 32 volatile compounds in abundance ranking and volatile compounds with significant differences in the four high-temperature Daqu samples.

HS-SPME-GC-MS identified 159 volatile compounds, including 22 nitrogen-containing heterocycles, 17 aldehydes, 23 ketones, 13 acids, 16 esters, 42 alcohols, 10 phenols, 3 ethers, and some other compounds, in the four studied Daqu types (Table S1). Among the four samples, MX had the highest total content of volatile compounds, which was approximately 1.5 times that of ZJ and ZS and 3 times that of SJ (Figure 2A). All four samples had relatively large amounts of alcohols, ketones, aldehydes, and nitrogen-containing heterocycles, accounting for 23.6–47.5%, 14.3–27.5%, 11.1–12.8%, and 6.6–17.0% of the total volatile compounds, respectively (Figure 2B). Among the alcohols, phenethyl alcohol had the highest average content, followed by 3-octanol. 2-Octanone was the predominant ketone. The main aldehydes were phenylacetaldehyde, benzaldehyde, and nonanal, and the main nitrogen-containing heterocyclic compounds were pyrazines, including tetramethylpyrazine and 2,3,5-trimethylpyrazine. The proportion of ester compounds was relatively low, and ethyl hexanoate was present in all four samples (Table S1). All of these substances were found in previous studies [26]. A Venn diagram showed the volatile compounds unique to and shared by each sample (Figure 2C). Among the volatile compounds, 34 were shared among the 5 samples, acting as their main components (Figure 2D). MX had the most types of unique volatile compounds, and ZS had the highest content of unique volatile compounds. The number of unique volatile compounds in each sample was significantly fewer than their shared volatile compounds (Figure 2D). Among the shared volatile compounds, phenethyl alcohol, isovaleric acid, 2-octanone, tetramethylpyrazine, 3-octanol, and phenylacetaldehyde were relatively abundant and were the main volatile compounds in each Daqu sample. PLS-DA analysis revealed that the differences in the composition of volatile compounds between ZJ and SJ were relatively small, and those between ZS and the other three samples were relatively large (Figure S3A). Meanwhile, six volatile compounds with statistical variability (VIP value > 1.0), namely, 2-octanone, phenethyl alcohol, 3-octanol, 2,5-dimethyl-3-butyl-pyrazine, isovaleric acid, and [R,(-)]-2-pentanol, were found to contribute significantly to the changes in the volatile characteristics of the four samples (Figure S3B). The contents of these volatile compounds in MX were relatively low (Figure 2E).

### 3.3. Analysis of Microbial Community Co-Occurrence Patterns and Microbial Biomarkers in High-Temperature Daqu

#### 3.3.1. Microbial Community Composition and Diversity

A total of 77.41 Gbps of raw bases were obtained from the four high-temperature Daqu samples (Table S2). The raw Q20 and Q30 of all samples were greater than 97% and 93%, respectively, indicating high sequencing quality and reliable data. After quality control, 75.89 Gbps of clean bases remained, with clean Q20 all greater than 98% and clean Q30 all greater than 95%. The clean bases were assembled into 534,798, 381,327, 251,623, and 290,512 contigs in ZS, MS, SJ, and ZJ, respectively, and all achieved relatively high N50 and N90 lengths (bp), indicating high assembly quality. A total of 2,010,973 ORFs were obtained through gene prediction, with 444,720, 529,064, 502,978, and 534,211 ORFs obtained from ZS, MX, SJ, and ZJ, respectively.

**Figure 3:**
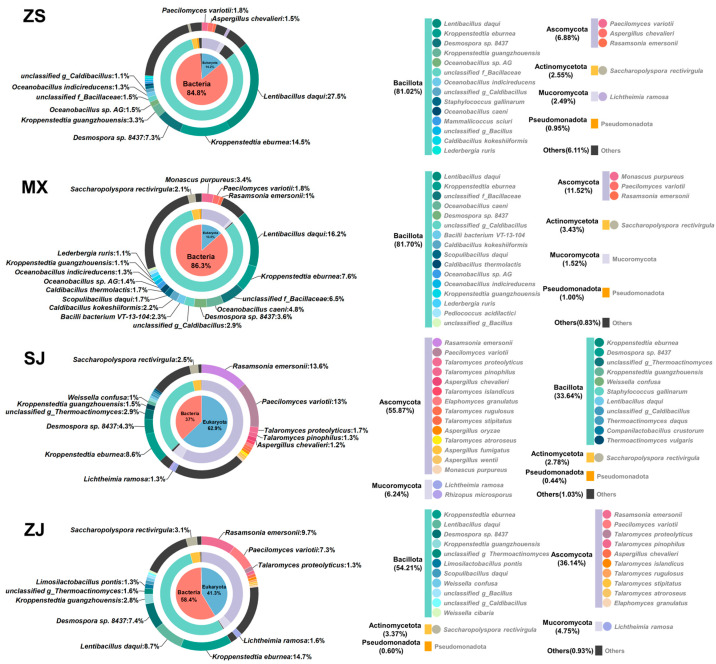
The variations in the microbial community of four high-temperature Daqu samples at the domain (inner circles), phylum (median circles), and species (outer circles) levels; only the species with a relative abundance more than 1.0% in each Daqu are displayed.

At the domain level, the four Daqu samples were all composed of prokaryotes, eukaryotes, archaea, and viruses. However, the proportions of archaea and viruses in all samples were less than 1%, similar to the findings in previous studies [9]. The proportions of ZS and MX at the domain level were relatively close, with prokaryotes being the dominant ones (84.8% and 86.3%, respectively). The proportion of prokaryotes in ZJ was lower than that in ZS and MX (58.4%), and eukaryotes were the dominant ones in SJ (62.9%) (Figure 3).

The prokaryotes in the four samples were mainly composed of Bacillota at the phylum level, accounting for 90.9–95.4% of the total prokaryotes, followed by Actinomycetota (3.0–7.5%) and Pseudomonadota (1.0–1.2%). At the species level, seventeen dominant bacteria (relative abundance > 1%) were found in the four samples, three of these dominant bacteria, namely, *Kroppenstedtia eburnean*, *Desmospora* sp. 8437, and *Kroppenstedtia guangzhouensis*, were shared among the four samples. MX had the largest number of dominant bacterial species (15) and the most unique dominant fungi (6), which might be the reason behind its lowest Simpson index and highest Shannon index (Figure S3). Different from MX, ZS and ZJ had higher Simpson indices and lower Shannon indices, indicating that a relatively small number of bacteria dominated their bacterial communities. This finding explained why ZS and ZJ had a similar proportion of bacterial abundance to MX, but the number of their dominant bacterial species (8) was far less than that of MX. Eukaryotes were mainly composed of Ascomycota at the phylum level, accounting for 48.8–88.84% of the total eukaryotes, followed by Mucoromycota (9.9–17.6%). At the species level, six dominant fungi were found, including *Paecilomyces variotii* that was shared among the four samples. According to the α-diversity indices, SJ had the highest eukaryotic richness (ACE index and Chao index) and diversity (Shannon index), probably because it had the largest proportion of relative abundance of eukaryotes. Meanwhile, there was no significant difference in the Simpson index of eukaryotes, indicating that the dominance degrees of the dominant fungi in the four samples were similar.

#### 3.3.2. Identifying Co-Occurrence Patterns in High-Temperature Daqu Microbial Communities by Network Analysis

**Figure 4:**
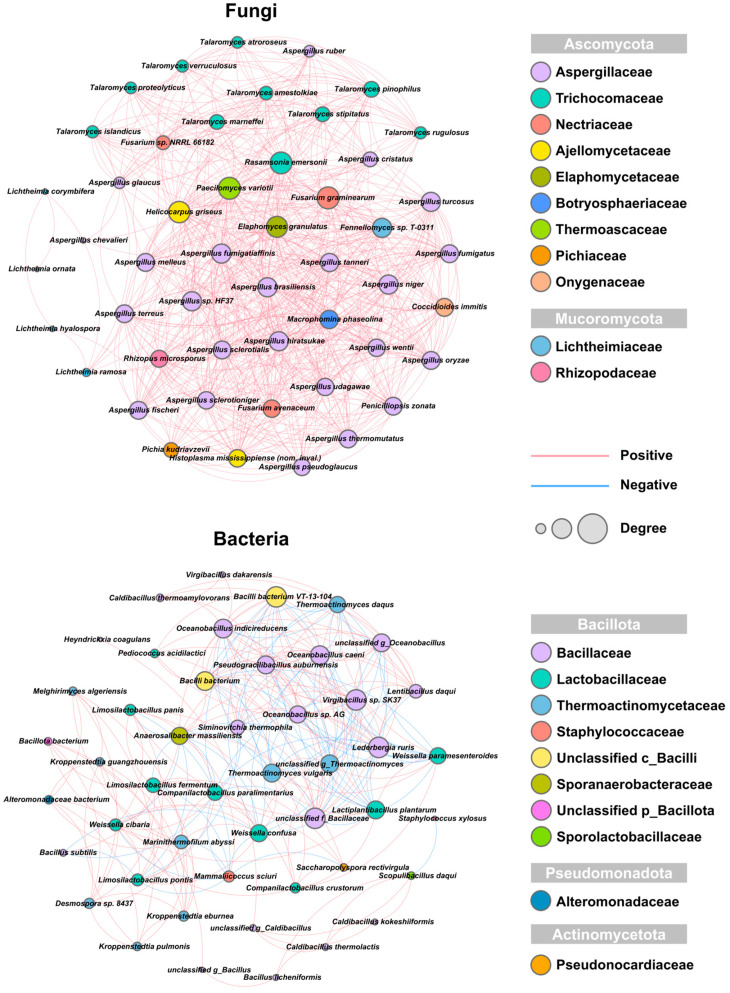
Co-occurrence networks of bacteria and fungi in high-temperature Daqu from Southern Sichuan–Northern Guizhou region based on Spearman correlation coefficients. Nodes represent individual microbial species. Red and blue edges represent significantly positive and negative correlations, respectively (Spearman’s r > 0.75 or < −0.75, *p* < 0.05).

We explored the co-occurrence and co-exclusion patterns of microbial communities based on the Spearman correlation coefficients (|r| > 0.75, *p* < 0.05) of 100 dominant species (50 dominant bacterial species and 50 dominant fungal species) to elucidate the interactions among microbial communities in different high-temperature Daqu samples (Figure 4). The fungal community obtained 49 nodes, slightly higher than that of the bacterial community (47 nodes). In addition, the number of edges, average degree, density, and average clustering coefficient of the fungal community were all greater than those of the bacterial community. Meanwhile, the proportion of positive correlations reached 100%, indicating that the interaction relationships among fungi were significant and had a significant co-occurrence pattern. Most fungal species within the genus *Ascomycota* had complex positive correlation relationships. Among them, 20 fungal species in the family *Aspergillaceae* and all fungal species in the family *Trichocomaceae* constituted two dense positive correlation networks. However, only four fungal species in the family *Lichtheimiaceae* of the genus *Mucoromycota* showed positive correlations with a small number of other fungi. *Aspergillus chevalieri* also showed the same situation with other fungi. The bacterial community had a large network diameter and path length and a 33.33% negative correlation relationship, indicating that the connections among bacteria were weak and exclusion was present. This finding suggested that the connections within the bacterial community were relatively loose and susceptible to the influence of external disturbances [12]. The exclusion in the bacterial community was mainly observed in three bacterial families (*Bacillaceae*, *Lactobacillaceae*, and *Thermoactinomycetaceae*) in the phylum Bacillota. Among them, 10 bacteria in the family *Bacillaceae*, *Bacilli bacterium* VT-13-104, and *Bacilli bacterium* constituted the densest positive correlation network in the bacterial community. This network showed negative correlations with four bacteria in the family *Lactobacillaceae* (*Weissella paramesenteroides*, *Lactiplantibacillus plantarum*, *Weissella confusa*, and *Companilactobacillus paralimentarius*) and three bacteria in the family *Thermoactinomycetaceae* (*Thermoactinomyces* (*T. Daqus* and *T. vulgaris*) and *unclassified g_Thermoactinomyces*). In addition to the above microorganisms, seven bacteria belonging to the family *Thermoactinomycetaceae* constituted a small co-occurrence set. Overall, our results indicated differences in network patterns between the fungal and bacterial communities in the four Daqu samples.

#### 3.3.3. Analysis of Shared and Unique Species and Recognition of Biomarkers

**Figure 5:**
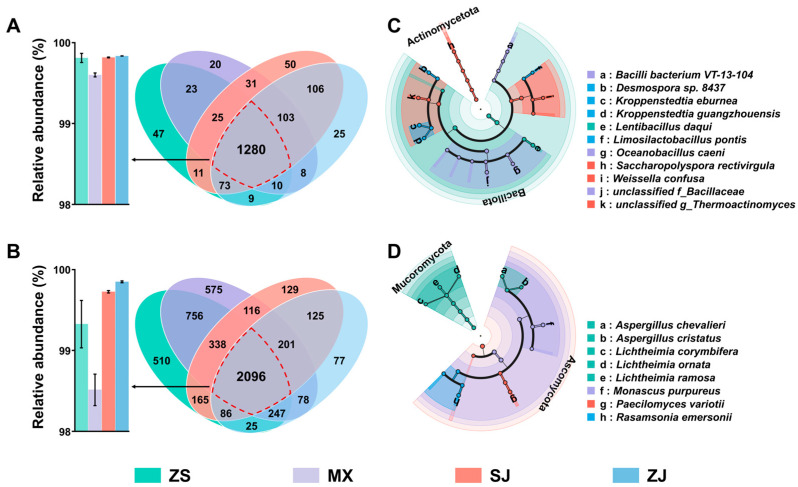
Venn and bar diagrams showing number of shared and unique (**A**) bacterial and (**B**) fungal species and corresponding relative abundance of shared microbial community. Linear discriminant analysis effect size (LEfSe) of bacterial communities (**C**) and fungal communities (**D**) among different Daqu samples (LDA score ≥ 4).

At the species level, the Venn diagram showed 1280 bacterial species shared among the four Daqu samples, with the abundances of the shared bacteria accounting for more than 99.60% in each sample (Figure 5A,B). A total of 2096 fungal species were shared among the four Daqu samples, and the abundances of the shared fungi accounted for 99.31%, 98.50%, 99.71%, and 99.83% in the four Daqu samples. Compared with SJ (129 bacteria) and ZJ (77 bacteria), ZS (510 bacteria) and MX (575 bacteria) had more unique bacteria. The number of unique bacteria in each sample was significantly greater than that of the unique fungi. LEfSe analysis was used to further explore the biomarkers in the four samples (Figure 5C,D). Six biomarkers were found in the ZS samples, including five fungi (*Aspergillus chevalieri*, *Lichtheimia ramosa*, *Lichtheimia ornata*, *Aspergillus cristatus*, and *Lichtheimia corymbifera*) and one bacterium (*Lentibacillus Daqui*). Different from those in ZS, the biomarkers in MX, SJ, and ZJ were mainly bacteria, including *unclassified f_Bacillaceae*, *Oceanobacillus caeni*, and *Bacilli bacterium* VT-13-104 in MX; *unclassified g_Thermoactinomyces*, *Saccharopolyspora rectivirgula*, and *Weissella confusa* in SJ; and *Kroppenstedtia eburnea*, *Desmospora* sp. 8437, *Kroppenstedtia guangzhouensis*, and *Limosilactobacillus pontis* in ZJ. In addition, one biomarker belonged to each of the fungal communities of MX, SJ, and ZJ, namely, *Monascus purpureus* for MX, *Paecilomyces variotii* for SJ, and *Rasamsonia emersonii* for ZJ. Overall, the biomarkers in each sample belonged to the shared microorganisms, mainly fungi from the families *Aspergillaceae* and *Thermoascaceae* and bacteria from the families *Bacillaceae*, *Thermoactinomycetaceae*, and *Lactobacillaceae*.

### 3.4. Correlation Among Microbial Community, Physicochemical Properties, and Volatile Compounds in High-Temperature Daqu

**Figure 6:**
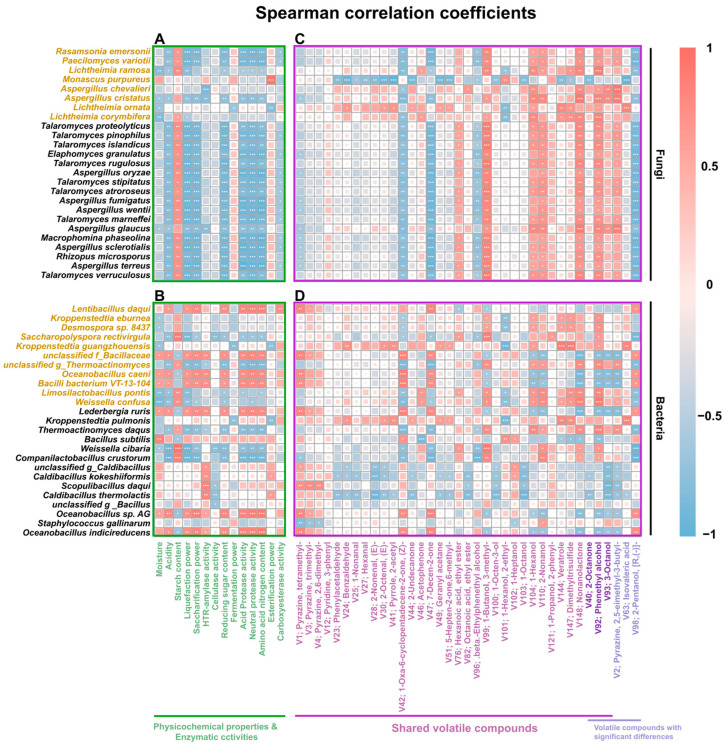
The correlation of 50 predominant microbial species (including the 20 top fungal species (**A**,**C**) and the 20 top bacterial species (**B**,**D**)) with physicochemical properties, enzymatic activities, and volatile compounds in high-temperature Daqu from the Southern Sichuan–Northern Guizhou region. Here, * indicates a significant correlation at the 0.05 level, ** at the 0.01 level, and *** at the 0.001 level. The microorganisms named with the gold color are biomarkers. The volatile compounds include 34 shared volatile compounds ranked by abundance and 6 volatile compounds with significant differences.

Spearman correlation analysis was conducted to explore the relationships between 14 physicochemical and enzyme activity indicators and the major microorganisms (including the top 25 fungal species and the top 25 bacterial species in relative abundance) to identify the microorganisms responsible for the differences in physicochemical indicators and enzyme activities among the four high-temperature Daqu samples (Figure 6A,B). In the fungal community, *Monascus purpureus* showed a significant positive correlation with esterifying power, further demonstrating the positive role of microorganisms of the *Monascus* genus in the synthesis of ester substances [27]. Meanwhile, no significant positive correlation was found between fungal species and starch/protein degradation indicators. The reason may be that the close interrelationships among fungi in the high-temperature Daqu from this region led to the co-evolution of a relatively universal pattern of starch/protein degradation. In the bacterial community, eleven bacteria, including four identified as biomarkers, showed a positive correlation with the indicators related to starch degradation. Among them, *Lentibacillus Daqui* showed a significant positive correlation with saccharification power and reducing sugar content, and *unclassified f_Bacillaceae*, *Oceanobacillus caeni*, and *Bacilli bacterium* VT-13-104 showed significant positive correlations with saccharification power, liquefaction power, heat-resistant amylase, and reducing sugar content. In addition, seven bacteria, including the above four bacterial biomarkers, did not show significant positive correlations with acidic/neutral protease activities and amino acid nitrogen content.

This study investigated the correlation between volatile compounds and microorganisms in high-temperature Daqu to identify the microorganisms responsible for volatile compound variations. Spearman correlation analysis was conducted on the top 34 shared volatile compounds and 5 compounds with different abundance and the major microorganisms (Figure 6C,D). Eight bacteria (including the above four bacterial biomarkers) showed positive correlations with the indicators related to starch and protein degradation and with three pyrazines (tetramethylpyrazine, trimethylpyrazine, and 2,6-dimethylpyrazine), two ketones (7-decen-2-one and (Z)-1-oxa-6-cyclopentadecene-2-one), and one alcohol ((R)-(-)-2-Pentanol). Two bacteria (*Kroppenstedtia (K. guangzhouensis and K. pulmonis)*) and one fungus (*Lichtheimia ornata*) showed positive correlations with six aldehydes (phenylacetaldehyde, benzaldehyde, 1-nonanal, hexanal, and (E)-2-nonenal), five ketones (2-undecanone, acetophenone, geranyl acetane, 5-hepten-2-one, 6-methyl-, and 2-octanone), and one acid (isovaleric acid). Most fungi and five bacteria (*unclassified g_Thermoactinomyces*, *Saccharopolyspora rectivirgula*, *Limosilactobacillus pontis*, *Weissella cibaria*, and *Thermoactinomyces Daqus*) showed positive correlations with one ester (ethyl hexanoate), five alcohols (3-methyl-1-butanol, 1-hexanol, 2-nonanol, phenethyl alcohol, and 3-octanol), and one pyrazine (2,5-dimethyl-3-butylpyrazine). These microorganisms may be responsible for the differences in volatile compounds among the four high-temperature Daqu samples.

### 3.5. Functional Gene Category by Blasting to COG, CAZy, KEGG Databases

#### 3.5.1. Functional Gene Annotation Based on COG and KEGG Databases

Metagenomic data were annotated using the KEGG and COG databases to explore the commonalities and differences in the functional characteristics of microbial communities and biomarkers among the four high-temperature Daqu samples.

**Figure 7:**
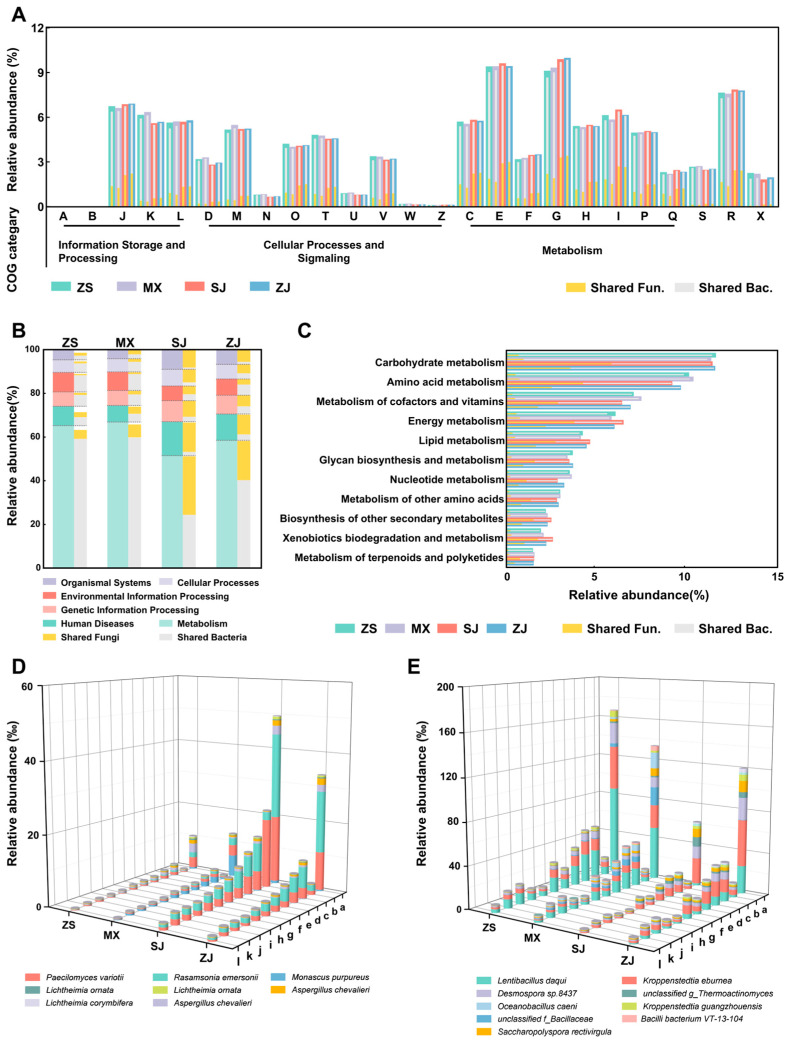
Functional annotation of Daqu microbiota genes by the COG (**A**) and KEGG (**B**,**C**) databases. The specific function description of each COG category is shown in Table S4. The functional annotations of fungal biomarkers (**D**) and bacterial biomarkers (**E**) in different Daqu samples in the KEGG database show the diverse functional contributions of various microorganisms to high-temperature Daqu. The specific functional description of each KEGG code is shown in Table S5.

According to the annotation results of the COG database, the number of functional genes belonging to the metabolism category was the highest (Figure 7A). Among the functional genes in the metabolism category, the relative abundances of the functional genes belonging to carbohydrate transport and metabolism (G) and amino acid transport and metabolism (E) were close and were the highest. This result indicated that the metabolism of carbohydrates and amino acids is the most important biological function in high-temperature Daqu, contributing to the normal succession of the microbial community and providing the basic saccharification function and significant aroma-formation function for high-temperature Daqu [14].

Similarly to the annotation results of the COG database, the genes related to metabolism were the most abundant in all Daqu microbiota at level 1 of the KEGG database. The proportions of these genes in ZS and MX (65.33% and 66.97%) were higher than those in SJ and ZJ (51.51% and 58.58%) (Figure 7B). Among them, the genes related to metabolism in ZS and MX were mainly provided by their shared bacteria, with proportions of 59.18% and 59.81%, respectively. Meanwhile, the abundance of the genes provided by the shared bacteria in SJ and ZJ was lower than that in the other two samples. In particular, the genes in SJ were mainly provided by the shared fungi (26.73%). We then analyzed the distribution of functional genes related to metabolism within level 2 of the KEGG pathways (Figure 7C). Except for global and overview maps, carbohydrate metabolism and amino acid metabolism were the two functional categories with the largest proportions in each Daqu sample, verifying the annotation results of the COG database and suggesting that high-temperature Daqu has great potential for substrate degradation and aroma-formation [28]. Meanwhile, the genes of the two functional categories in each sample were relatively close (12.64–12.92%, 10.23–11.54%). Similarly to the situation in level 1 pathways, the genes of the two functional categories in ZS and MX were mainly provided by their shared bacteria (carbohydrate metabolism: 11.96% and 11.35%; amino acid metabolism: 10.40% and 10.55%). The shared fungi in SJ and ZJ provided more of the genes of the two functional categories compared with those in ZS and MX.

The metabolic functional genes of biomarkers in each Daqu sample were annotated according to level 2 of the KEGG database. Among the fungal biomarkers, the ones that mainly provided genes related to metabolism for SJ and ZJ were *Paecilomyces variotii* and *Rasamsonia emersonii*. *Paecilomyces variotii* also provided genes related to glycan biosynthesis and metabolism for SJ, which were significantly higher in SJ than in the other samples (Figure 7D). Meanwhile, the total abundances of the genes related to metabolism provided by all fungal biomarkers for ZS and MX were fewer than those of SJ and ZJ, but *Monascus purpureus* provided more metabolic functional genes for MX than for the other three samples. Among the bacterial biomarkers, *Kroppenstedtia eburnean* and *Desmospora* sp. 8437 had substantial contributions to the genes related to metabolism in the four samples. However, the abundances of the genes related to metabolism provided by the remaining microorganisms in the four samples were different. The genes provided by *Lentibacillus Daqui* were more in ZS, MX, and ZJ than in SJ, those provided by *Saccharopolyspora rectivirgula* were the fewest in ZS, those provided by *Unclassified f_Bacillaceae* were the most in MX, and those provided by *Kroppenstedtia guangzhouensis* were more in ZJ and ZS than in MX and SJ. Among these bacterial biomarkers, *unclassified f_Bacillaceae*, *Kroppenstedtia eburnean*, *Desmospora* sp. 8437, and *Saccharopolyspora rectivirgula* contributed greatly to amino acid metabolism (Figure 7E).

#### 3.5.2. Prediction of Microbial Functions in Daqu Based on KEGG Database

**Figure 8:**
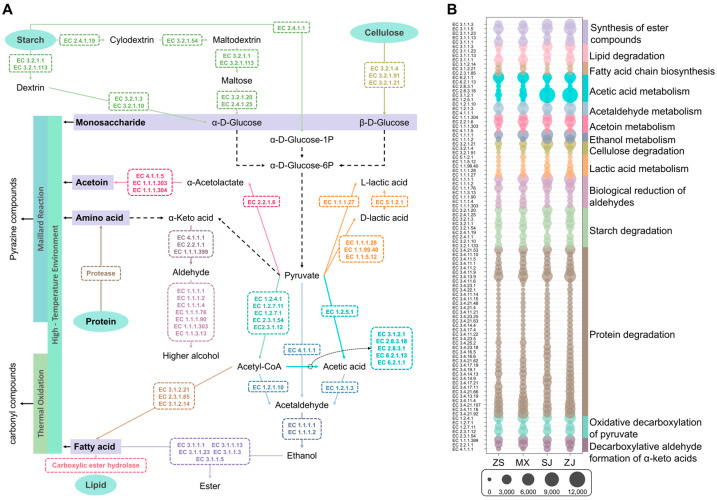
Functional prediction of microorganisms in four high-temperature Daqu samples. (**A**) Primary metabolic pathways involved in degradation of raw materials and formation of flavor compounds. (**B**) Abundance and functional classification of main enzymes.

To further explore how microorganisms affect the saccharification, fermentation, and aroma-formation in high-temperature Daqu, we integrated the metabolic pathways most relevant to these processes in high-temperature Daqu based on the KEGG database. We then predicted 97 enzymes that might be involved in substrate degradation and aroma-formation in high-temperature Daqu and categorized them into 14 functional parts (Figure 8A,B). Nine enzymes that might be involved in the starch degradation pathway were predicted, and they jointly ensured the saccharification power of high-temperature Daqu. Among them, α-amylase (EC 3.2.1.1), 1,2-α-mannosidase (EC 3.2.1.113), glycogen phosphorylase (EC 2.4.1.1), and cyclomaltodextrin glucanotransferase (EC 2.4.1.19) also provided liquefaction power for high-temperature Daqu. In addition, three enzymes that might be involved in the cellulose degradation pathway were predicted, with β-glucosidase (EC 3.2.1.21) as the main cellulose-degrading enzyme. This finding was consistent with previous studies [29]. Amylase abundance did not consistently correlate with saccharification, liquefaction, or high-temperature amylase activity; similarly, cellulase abundance and activity showed an inconsistent relationship. This discrepancy may be attributed to the post-translational modifications of enzymes, the interactions among enzymes, and the limitations of databases.

Starch and cellulose are degraded into reducing sugars under the action of above functional enzymes and then produce pyruvate via the glycolytic pathway. In this study, 21 functional enzymes were predicted and found to be involved in pyruvate metabolism to lactic acid, acetic acid, acetaldehyde, and ethanol. Alcohol dehydrogenase (EC 1.1.1.1) exhibited high abundance across all the Daqu samples and may be crucial for the fermentation power of high-temperature Daqu [9]. Pyruvate is also an important precursor for various alcohols, pyrazines, acids, esters, aldehydes, and ketones. In this study, four enzymes were predicted to be involved in acetoin metabolism, which can help convert pyruvate into pyrazine [30]. Moreover, the four Daqu samples from the Sichuan–Guizhou region all contained similar abundances of acetoin-metabolizing enzymes, which can ensure pyrazine synthesis. Pyruvate can be converted into α-keto acids through transaminases, pyruvate carboxylase, and oxidative decarboxylation reactions and then into higher alcohols through the decarboxylation and aldehyde group formation of α-keto acids and the biological reduction in aldehydes [14]. Seven aldehyde reductases were predicted. In the four Daqu samples, the abundance of alcohol dehydrogenase and alcohol dehydrogenase (NADP+) (EC 1.1.1.2) was positively correlated with the level of phenethyl alcohol. Similarly, the abundance of (R,R)-butanediol dehydrogenase (EC 1.1.1.4) was positively correlated with the level of 3-octanol. Meanwhile, three enzymes were predicted to convert acetyl-CoA (obtained from the oxidative decarboxylation of pyruvate) into fatty acids, providing substrates for esterification and fatty acid oxidation. In the four Daqu samples, the abundance of the fatty acid synthase system (EC 2.3.1.85) was positively correlated with the level of 7-decen-2-one and (Z)-1-oxa-6-cyclopentadecene-2-one [9]. In addition, 38 proteases were predicted, with Xaa-Pro dipeptidase (EC 3.4.13.9), endopeptidase Clp (EC 3.4.21.92), methionyl aminopeptidase (EC 3.4.11.18), peptidase Do (EC 3.4.21.107), and endopeptidase La (EC 3.4.21.53) being the dominant ones. These proteases generate free amino groups that participate in the Maillard reactions with carbonyl compounds, contributing to pyrazine formation in high-temperature Daqu [26]. Four carboxylic ester hydrolases, namely, carboxylesterase (EC 3.1.1.1), sterol esterase (EC 3.1.1.13), acylglycerol lipase (EC 3.1.1.23), and triacylglycerol lipase (EC 3.1.1.3), were predicted and found to accelerate the synthesis of aldehydes, ketones, and esters in high-temperature Daqu via lipid degradation.

#### 3.5.3. Investigation into Starch Degradation Capacity of Daqu Based on CAZy Database

**Figure 9:**
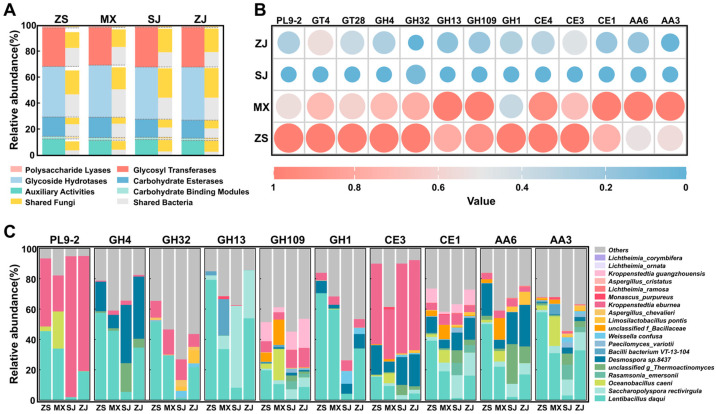
(**A**) Annotation of carbohydrate enzymes in Daqu microbiota at class level through CAZy database. (**B**) Abundance differences in enzyme families that are correlated with starch degradation ability of four high-temperature Daqu samples. (**C**) Traceability analysis of key enzyme families.

Although the saccharification ability of high-temperature Daqu is significantly lower than that of low-, medium-, and medium–high-temperature Daqu due to production processes and requirements [11], the abundance of its functional genes involved in carbohydrate metabolism is still the highest. Therefore, we explored the commonalities and differences in the carbohydrate utilization ability among the four Daqu samples using the CAZy database to further analyze the relationship between amylases and starch degradation capacity in high-temperature Daqu from the Southern Sichuan–Northern Guizhou region. At the class level of the CAZy database, six functional categories were annotated. Among them, glycoside hydrotases (39.00–41.15%) and glycosyl transferases (29.00–30.56%) dominated in all the samples, which was similar to the research results of Yang [31]. Glycoside hydrotases are an important group of enzymes that hydrolyze glycosidic bonds, including the enzymes associated with starch liquefaction, glycation, and cellulose degradation [14]. As is consistent with the KEGG database annotations, the CAZy database analysis indicated that the shared microorganisms primarily encode the functional genes in each category (Figure 9A).

Kruskal–Wallis H test identified 244 carbohydrate enzyme families with significant intersample variation (*p* < 0.05) across the four Daqu samples. The top 50 most abundant families (Figure S4) were analyzed, revealing 13 families correlated with starch degradation capacity (Figure 9B). These families included five glycoside hydrolases, namely, GH4, GH32, GH13, GH109, and GH1. GH13 is mainly related to starch degradation and can hydrolyze α-1,4-glycosidic bonds and α-1,6-glycosidic bonds, directly participating in starch decomposition. The other glycoside hydrolases mainly act on galactose, sucrose, and cellobiose. In addition to the above five glycoside hydrolases, some other enzyme families were correlated with the starch degradation capacity, such as PL9-2 (pectin degradation), CE3 (lignocellulose degradation), CE1 (hemicellulose degradation), and AA6/AA3 (carbohydrate redox reactions). Overall, the correlation of these enzyme families other than GH13 with the starch degradation capacity may be explained by their indirect contribution to starch degradation or their ability to synergistically enhance GH13 activity.

Traceability analysis indicated that the GH13 metabolized by *Lentibacillus Daqui* was the main reason for the intersample variation in GH13 abundance and its correlation with starch degradation capacity. Other enzyme families’ variations in abundance and their correlations with the starch degradation capacity were primarily attributed to the metabolisms of *Lentibacillus Daqui*, *Kroppenstedtia eburnean*, and *Desmospora* sp. 8437. This phenomenon indicated the important positions of these three microorganisms, particularly *Lentibacillus Daqui*, in starch degradation via the combined action of multiple enzymes produced by their metabolisms.

## 4. Discussion

This study investigated the microbial community and function of four high-temperature Daqu samples in the Southern Sichuan–Northern Guizhou region to elucidate the factors underlying their diverse characteristics.

Comparative analysis revealed shared bacterial taxa across the four Daqu samples. At the species level, *Kroppenstedtia eburnean*, *Desmospora* sp. 8437, and *Kroppenstedtia guangzhouensis* were dominant in the four Daqu samples (Figure 3). These three bacteria are all heat-resistant members of the family *Thermoactinomycetaceae*, and their dominant status may be due to their enrichment in high-temperature environments [32]. Compared with regions such as Xiangyang in Hubei Province [22,33], Beijing [34], Hechi in Guangxi Province [25], and Sanmenxia in Henan Province [35], only the Southern Sichuan–Northern Guizhou region exhibits a situation where *Desmospora* sp. 8437 shows a dominant proportion in the high-temperature Daqu. This phenomenon may be one reason for the differences in Daqu samples across various regions. However, further research is needed to determine the causal relationship. Moreover, despite the variations in microbial community composition revealed by microbial community composition analysis, functional gene analysis revealed remarkable consistency in overall metabolic functions across the four Daqu samples (Figure 7A–C), indicating functional redundancy [36] that possibly contributed to the consistent performance of high-temperature Daqu [37].

Analysis of the saccharification and aroma-producing capabilities of four Daqu samples further elucidated the similarities and differences in the functional roles of microorganisms within high-temperature Daqu from the Southern Sichuan–Northern Guizhou region. Starch and protein degradation by the Daqu microbiome significantly influences the saccharification and aroma-formation functions of Daqu and the subsequent Baijiu brewing [38]. Analysis of physicochemical properties and enzymatic activities indicated that ZS and MX exhibited superior starch and protein degradation capabilities among the four Daqu samples (Figure 1). Correlation analysis identified several key bacterial contributors to starch and protein degradation: *Oceanobacillus caeni* and *Bacilli bacterium* VT-13-104 (MX), an *unclassified f_Bacillaceae* (MX), and *Lentibacillus Daqui* (ZS) (Figure 6). Most of the identified bacteria belonged to the family *Bacillaceae* [39]. Functional gene analysis revealed a correlation between the abundance of carbohydrate and amino acid metabolism genes encoded by these bacterial species and the starch and protein degradation capabilities of Daqu (Figure 7D,E). This correlation explained the greater saccharification, liquefaction, protease activity, and amino acid nitrogen content observed in ZS and MX compared with those in SJ and ZJ. Among the four Daqu samples, MX exhibited the highest abundance of three heat-resistant and thermophilic bacteria (*Oceanobacillus caeni*, *Bacilli bacterium* VT-13-104, and *Scopulibacillus Daqui*) (Figure 6) [40,41], potentially explaining its highest HTR-amylase activity. This higher HTR-amylase activity likely promotes starch degradation during subsequent high-temperature fermentation in Jiang-flavor Baijiu production.

Predictive analysis identified 9 enzymes involved in starch degradation and 38 enzymes involved in protein degradation based on the KEGG database (Figure 8). These findings largely agreed with a previous metagenomic study [38]. However, the high abundance of oligo-1,6-glucosidase was not observed in previous metaproteomic works [6,7,42]. This phenomenon indicated a certain deviation in using metagenomics alone to predict enzymes. Metagenomics reflect gene potential rather than actual expression, which is accurately captured by metaproteomics [43]. The observed differences underscored the importance of integrating multiple omics approaches to comprehensively understand enzymes and their contributions to Daqu fermentation and Baijiu brewing. In addition, we further analyzed the carbohydrate enzyme families related to the starch degradation capacity through the CAZy database. The results showed that the GH13 metabolized by *Lentibacillus Daqui* was correlated with starch degradation capacity, which might explain the observed differences in starch degradation capacity among the four high-temperature Daqu samples (Figure 9).

The analysis of volatile compounds focused on pyrazines, aldehydes, ketones, alcohols, and esters. Pyrazines contribute greatly to the roasty, nutty, and baked aromas of Baijiu, which are fundamental to the mellow flavor profile of Jiang-flavor Baijiu. Therefore, the abundance of pyrazines in Daqu and the ability to metabolically produce pyrazines are crucial for the quality of Jiang-flavor Baijiu [26]. Although the acetoin pathway was implicated in pyrazine production [44], this study found no correlation between key enzymes in this pathway and pyrazine levels (Figure 8B). This finding suggested that other mechanisms, such as the Maillard reaction, may account for the observed variations in the four high-temperature Daqu samples from the Southern Sichuan–Northern Guizhou region [45]. Microorganisms may influence pyrazine abundance by modulating the types and concentrations of reactants (carbonyl compounds and free amino groups) involved in the Maillard reaction within high-temperature Daqu. ZS and MX exhibited higher levels of several shared pyrazines (tetramethylpyrazine, trimethylpyrazine and 2,6-dimethylpyrazine) than SJ and ZJ (Figure 2E). This trend is potentially due to the presence of many amino groups and carbonyl compounds in ZS and MX resulting from the high activity of four biomarkers, namely, *unclassified f_Bacillaceae*, *Oceanobacillus caeni*, and *Bacilli bacterium* VT-13-104 in MX and *Lentibacillus Daqui* in ZS [46,47]. SJ showed a high level of 2,5-dimethyl-3-butylpyrazine, possibly because *Paecilomyces variotii* provided many genes related to glycan biosynthesis and metabolism (Figure 7D), which further affected the types and contents of carbonyl compounds [48]. The Maillard reaction is inherently complex, and the intricate chemical composition of Daqu further compounds this complexity. The microorganisms in Daqu represent only one factor influencing the Maillard reaction. A more comprehensive understanding of pyrazine production requires a systematic investigation of the Maillard reaction in Daqu, integrating data on environmental parameters (e.g., temperature and humidity) and the reaction kinetics.

Aldehydes and ketones, which play a balancing role in Daqu flavor [40], also greatly influence the aroma profile of Jiang-flavor Baijiu. The aldehydes and ketones in Daqu are primarily formed through non-enzymatic reactions during fermentation [26], and their abundance is often positively correlated with temperature [45]. In this study, the correlation between two volatile compounds (7-decen-2-one and (Z)-1-oxa-6-cyclopentadecene-2-one) and the microorganisms was consistent with the correlation observed between the shared pyrazines and the microorganisms (Figure 6). Hence, 7-decen-2-one and (Z)-1-oxa-6-cyclopentadecene-2-one may be products of the Maillard reaction. Most of the aldehydes and ketones were positively correlated with the abundance of *Kroppenstedtia (K. guangzhouensis and K. pulmonis)* and *Lichtheimia ornata* (Figure 6). This correlation is consistent with the known role of the thermal oxidation of unsaturated fatty acids in the formation of these compounds [49,50]. The heat-resistant *Kroppenstedtia* spp. [33] contributes genes involved in lipid metabolism (including carboxylesterase, triacylglycerol lipase, and sterol esterase), potentially accelerating the release of fatty acids and providing substrates for thermal oxidation [51]. *Lichtheimia ornata*, although not heat-resistant, may contribute to fatty acid production during the initial phases of fermentation (through the fatty acid synthase system), providing substrates for the later formation of aldehydes and ketones [52].

Higher alcohols contribute fresh and fruity aromas, and appropriate concentrations enhance the complexity and richness of the aroma profiles of Jiang-flavor Baijiu. Phenethyl alcohol and 3-octanol were correlated with multiple microbial taxa (Figure 6), suggesting a complex biosynthetic process involving multiple species. These higher alcohols are produced via α-keto acid metabolism, followed by deacetalization and aldehyde reduction [4,53]. Enzymes involved in α-keto acid deacetalization and aldehyde reduction were also identified (Figure 8A). Comparative analysis revealed a correlation between the abundance of aldehyde reductases and the levels of phenethyl alcohol and 3-octanol (Figure 8B). In particular, alcohol dehydrogenase and alcohol dehydrogenase (NADP+) were strongly correlated with phenylacetaldehyde levels, and (R,R)-butanediol dehydrogenase was correlated with 3-octanol.

Ethyl hexanoate imparts a complex aroma to Baijiu, similar to that of pineapple, mud, pickled cabbage, and plant ash [54]. Ethyl hexanoate production, resulting from the esterification of hexanoic acid and ethanol, varied across the Daqu samples. This variation was correlated with the abundance of lipid metabolism genes from *Saccharopolyspora rectivirgula* (SJ) and *Weissella confusa* (SJ) and *Limosilactobacillus pontis* (ZJ) (Figure 7E), potentially increasing the hexanoic acid levels. The high abundance of alcohol dehydrogenase in high-temperature Daqu ensured sufficient ethanol levels (Figure 8B) [55]. These bacteria also contributed enzymes such as lysophospholipase (EC 3.1.1.5) and triacylglycerol lipase, potentially enhancing ethyl hexanoate synthesis through accelerated esterification (Figure 8B). These findings are consistent with a previous report indicating the positive role of *Saccharopolyspora* spp. in ethyl hexanoate production [56].

Analysis of the microbial community co-occurrence network (Figure 4) revealed that four key species positively correlated with starch and protein degradation and shared pyrazine production had formed a network with nine additional *Bacillaceae* species. Similarly, two species positively correlated with aldehyde and ketone production had formed a network with five *Thermoactinomycetaceae* species. The species associated with 2,5-dimethyl-3-butylpyrazine, phenethyl alcohol, 3-octanol, and ethyl hexanoate did not exhibit strong co-occurrence patterns, indicating a complex biosynthetic mechanism for these compounds. Some bacteria showed a negative correlation with the *Bacillaceae*-dominated network [57]. These complex microbial interactions may greatly influence the physicochemical properties, enzyme activities, and volatile compound levels of high-temperature Daqu [13]. However, the specific influences require further research.

## 5. Conclusions

The structure and function of microbial communities in four high-temperature Daqu samples from the Southern Sichuan–Northern Guizhou region were analyzed using metagenomics. The influences of microbial communities on the physicochemical properties, enzyme activities, and volatile compounds of Daqu were then investigated. Results indicated that the microbial communities exhibited functional redundancy, contributing to the consistent performance of high-temperature Daqu. The dominance of *Desmospora* sp. 8437 was identified as a microbial characteristic unique to this region. The amylases and proteases metabolized by the family *Bacillaceae* were responsible for the differences in starch and protein degradation capabilities among the four Daqu samples. In particular, *Lentibacillus Daqui* influenced the starch degradation capacity across the four Daqu samples via GH13 metabolized by it. Moreover, the amylases metabolized by *Oceanobacillus caeni* and *Bacilli bacterium* VT-13-104 have strong high-temperature resistance. During Daqu production, the abundance of these bacteria can be modulated through environmental adjustments or by employing fortified Daqu, thereby enabling the regulation of the starch and protein degradation capabilities of Daqu. Variations in *Bacillaceae* abundance among the four Daqu samples influenced their levels of free amino groups and carbonyl compounds, thereby affecting the extent of the Maillard reaction and resulting in variations in common pyrazines and some ketones. The enzymes for lipid degradation metabolized by *Kroppenstedtia (K. guangzhouensis and K. pulmonis)* and *Lichtheimia ornata* affected the levels of most aldehydes and ketones. Multiple microorganisms contributed to the synthesis of alcohols and esters, influencing alcohol levels via aldehyde reductases and ethyl hexanoate levels via lipid metabolism enzymes and esterases. Overall, the differences in bacterial communities were the main cause of the diversities in saccharification and aroma production functions among the four high-temperature Daqu samples. This study provided a data basis for understanding the diversity of high-temperature Daqu samples in the Southern Sichuan–Northern Guizhou region. However, due to the complexity of the biochemical system within high-temperature Daqu, the underlying mechanisms of microbial interactions, the synthetic pathways of volatile compounds, and their interrelationships remain unclear. Therefore, a focus of future research should be the analysis of bacterial interaction networks to better elucidate cooccurrence patterns within the microbial community. Furthermore, a systematic investigation of non-enzymatic reactions in high-temperature Daqu is warranted, ultimately contributing to strategies for optimizing Daqu quality and flavor control.

## Data Availability

The original contributions presented in this study are included in the article/Supplementary Materials. Further inquiries can be directed to the corresponding author.

## Supplementary Materials

The following supporting information can be downloaded at: https://www.mdpi.com/article/10.3390/foods14040570/s1. Table S1: Main flavor substances detected in four high-temperature Daqu samples from Southern Sichuan–Northern Guizhou region; Table S2: Statistics of metagenomic sequencing and bioinformatics analysis; Table S3: Results of LDA discrimination analysis at species level of microbial community; Table S4: Description of each COG category; Table S5: Description of each KEGG code; Figure S1: Geographical distribution of high-temperature Daqu in Southern Sichuan–Northern Guizhou region; Figure S2: Score plot and loading plot of principal component analysis (PCA) reveal varied physicochemical profiles among four high-temperature Daqu samples; Figure S3: Ordination plot (A)and VIP plot (B) of partial least-squares discriminant analysis (PLS-DA) reveal volatile compounds with significant differences in four high-temperature Daqu samples; Figure S4: Differences in α-diversity indices of microbial communities among four high-temperature Daqu samples; Figure S5: Kruskal–Wallis H test bar plot of top 50 differential enzyme families in terms of abundance among four high-temperature Daqu samples.
