# Supplementary material for: Revealing the Differences in Microbial Community and Quality of High-Temperature Daqu in the Southern Sichuan–Northern Guizhou Region
Source: Foods. 2025 Feb 8;14(4):570. doi: 10.3390/foods14040570 (PMC11853950; doi:10.3390/foods14040570)
Supplement: Supplementary file 1 [file foods-14-00570-s001.zip › foods-3418776-supplementary.pdf]

Supplementary Materials

Revealing the Differences in Microbial Community and Quality of High-Temperature Daqu in the Southern Sichuan–Northern Guizhou Region

Cheng Yan[1], Zhangjun Huang[2, 3], Rongkun Tu[2, 3], Liqiang Zhang[2, 3], Chongde Wu[1], Songtao Wang[2, 3], Ping Huang[1], Yunhang Zeng[1,*], Bi Shi[1]

1 College of Biomass Science and Engineering, Sichuan University, Chengdu 610065, China
2 National Engineering Research Center of Solid-state Brewing, Luzhou 646000, China
3 Luzhou Laojiao Co., Ltd., Luzhou 646000, China

*** The complete contact details of the corresponding author:**

Name: Yunhang Zeng;
E-mail address: zengyunhang@scu.edu.cn
Tel./fax: +86-28-85405508;
Mailing address: College of Biomass Science and Engineering, Sichuan University, Chengdu 610065, China.

Document Statistics of Supplementary Materials:

Total pages: 18
Number of Figures: 5
Number of Tables: 5

Table S1. Main flavor substances detected in four high-temperature Daqu samples from the southern Sichuan–northern Guizhou region.

No.	Volatile compounds	CAS Number	Average concentration (ng/g dry weight)			
			**ZS**	**MS**	**SJ**	**ZJ**
	**Total volatiles**		**1219.89**	**428.47**	**811.17**	**843.80**
	**N-heterocyclic compounds**		**80.00**	**72.92**	**102.33**	**87.04**
V1	Pyrazine, tetramethyl-	1124-11-4	34.41	35.89	16.47	30.25
V2	Pyrazine, 2,5-eimethyl-3-butyl-	40790-29-2	18.38	nd	60.03	16.89
V3	Pyrazine, trimethyl-	14667-55-1	13.68	14.10	8.11	14.04
V4	Pyrazine, 2,6-dimethyl-	108-50-9	4.61	4.62	2.50	5.40
V5	Pyrazine, 2-ethyl-3,5,6-trimethyl-	17398-16-2	4.67	1.79	nd	5.20
V6	Pyrazine, 3,6-eimethyl-2-propyl-	18433-97-1	nd	nd	7.68	nd
V7	Pyrazine, 2,5-dimethyl-	123-32-0	nd	nd	nd	5.75
V8	Pyrazine, 2-methyl-6-vinyl-	13925-09-2	nd	nd	nd	2.96
V9	Pyrazine, 2,3-dimethyl-6-ethyl-	15707-34-3	1.33	1.48	nd	nd
V10	Pyrazine, 2-methyl-	109-08-0	nd	nd	nd	0.73
V11	Pyrazine, 3-ethyl-2,5-dimethyl-	13360-65-1	nd	nd	nd	0.68
V12	Pyridine, 3-phenyI	1008-88-4	2.06	2.29	2.34	2.31
V13	Pyridine, 2-acetyl-	1122-62-9	nd	2.23	nd	nd
V14	Pyridine, 4-hydroxy	626-64-2	nd	nd	1.68	nd
V15	Pyridine, 2,3-dihydroxy	16867-04-2	nd	nd	1.68	nd
V16	Pyridine, 3-(prop-2-yn-1-yloxy)	7223-42-9	nd	1.35	nd	nd
V17	Pyridine, 2-methyl-6-propyl-	5397-28-4	nd	0.88	nd	nd
V18	Pyridine, 2-propyl	622-39-9	nd	nd	nd	0.25
V19	Pyrrole, 2-acetyl-1-methyl	932-16-1	nd	5.06	nd	nd
V20	1H-Pyrazole, 4,5-dihydro-3-methyl-1-(2-methylpropyl)-	26964-53-4	nd	nd	1.85	2.57
V21	4,5-Pyrimidinediamine, 6-methoxy-	73318-76-0	nd	3.23	nd	nd

Table S1 (Continued 1). Main flavor substances detected in four high-temperature Daqu samples from the southern Sichuan–northern Guizhou region.

No.	Volatile compounds	CAS Number	Average concentration (ng/g dry weight)			
			**ZS**	**MS**	**SJ**	**ZJ**
V22	Pyridazine, 8-methyl-[1,2,4]triazolo[4,3-b]-	23069-75-2	0.87	nd	nd	nd
	**Aldehydes**		**148.14**	**54.87**	**90.03**	**98.18**
V23	Phenylacetaldehyde	122-78-1	45.63	20.71	24.29	23.68
V24	Benzaldehyde	100-52-7	26.15	13.66	15.54	20.90
V25	1-Nonanal	124-19-6	35.98	10.65	13.09	12.23
V26	2-Formylpyrrole	1003-29-8	8.40	nd	12.91	11.27
V27	Hexanal	66-25-1	10.29	5.84	6.90	9.07
V28	2-Nonenal, (E)-	18829-56-6	13.47	0.80	3.48	2.96
V29	N-Methylpyrrole-2-carboxaldehyde	1192-58-1	3.62	nd	3.43	5.37
V30	2-Octenal, (E)-	2548-87-0	2.38	0.47	1.65	1.97
V31	2-Phenyl-2-Butenal, (E)+(Z)	4411-89-6	1.13	nd	1.59	2.46
V32	2-Furaldehyde	98-01-1	nd	nd	1.91	2.86
V33	2-Heptenal, (E)-	18829-55-5	nd	nd	3.88	nd
V34	Vanillin	121-33-5	nd	nd	1.37	1.93
V35	2-Hexenal, 5-methyl-2-(1-methylethyl)-	35158-25-9	1.09	nd	nd	1.22
V36	4-Methyl-2-Phenyl-2-Pentenal, (E)+(Z)	26643-91-4	nd	nd	nd	1.67
V37	Benzaldehyde, 2-hydroxy-4-methyl-	698-27-1	nd	1.63	nd	nd
V38	Benzaldehyde, 3,4-dimethyl-	5973-71-7	nd	1.12	nd	nd
V39	Furfural, 5-methyl-	620-02-0	nd	nd	nd	0.58
	**Ketones**		**244.39**	**117.80**	**122.09**	**121.02**
V40	2-Octanone	111-13-7	101.16	41.97	57.38	50.90
V41	Pyrrole, 2-acetyl	1072-83-9	38.21	16.85	18.06	29.13
V42	1-Oxa-6-cyclopentadecene-2-one, (Z)-	63958-52-1	21.18	25.50	3.42	3.84

Table S1 (Continued 2). Main flavor substances detected in four high-temperature Daqu samples from the southern Sichuan–northern Guizhou region.

No.	Volatile compounds	CAS Number	Average concentration (ng/g dry weight)			
			**ZS**	**MS**	**SJ**	**ZJ**
V43	Acetone	67-64-1	9.13	nd	11.42	11.38
V44	2-Undecanone	112-12-9	10.52	4.40	6.39	6.19
V45	6-Undecen-2-one, (Z)-	107853-70-3	26.10	nd	nd	nd
V46	Acetophenone	98-86-2	3.81	3.24	3.86	9.08
V47	7-Decen-2-one	35194-33-3	9.12	3.61	1.55	2.67
V48	3-Octanone	106-68-3	4.07	nd	6.43	2.22
V49	Geranyl acetane	3796-70-1	5.64	1.67	2.28	2.33
V50	3(2H)-Furanone, 4-methoxy-2,5-dimethyl-	4077-47-8	nd	10.01	nd	nd
V51	5-Hepten-2-one,6-methyl-	110-93-0	3.25	1.15	1.35	1.77
V52	2-Heptanone	110-43-0	3.60	2.81	nd	nd
V53	D-Camphor	464-49-3	nd	nd	6.40	nd
V54	1,3-Benzodioxol-2-one, hexahydro-	4389-22-4	3.73	nd	nd	nd
V55	2-Tridecanone	593-08-8	nd	3.05	nd	nd
V56	1-Octen-3-one	4312-99-6	2.39	nd	0.49	nd
V57	Fitone	502-69-2	nd	nd	1.36	1.50
V58	2-Piperidone	675-20-7	2.48	nd	nd	nd
V59	2-Heptanone,6-methyl-	928-68-7	nd	nd	1.69	nd
V60	2-Butanone, 3-hydroxy-3-methyl-	115-22-0	nd	1.50	nd	nd
V61	Acetophenone, 2,4-dimethoxy	829-20-9	nd	1.09	nd	nd
V62	2-Pentadecanone	2345-28-0	nd	0.97	nd	nd
	**Acids**		**380.12**	**5.80**	**43.98**	**134.12**
V63	Isovaleric acid	503-74-2	238.50	nd	41.48	117.24
V64	Isobutyric acid	79-31-2	29.23	nd	nd	16.88

Table S1 (Continued 3). Main flavor substances detected in four high-temperature Daqu samples from the southern Sichuan–northern Guizhou region.

No.	Volatile compounds	CAS Number	Average concentration (ng/g dry weight)			
			**ZS**	**MS**	**SJ**	**ZJ**
V65	2-Pentenoic acid, 2,3-dimethyl-	122630-51-7	19.12	nd	nd	nd
V66	Pentanoic acid, 2,4-dimethyl-	5868-33-7	18.33	nd	nd	nd
V67	Heptanoic acid	111-14-8	17.37	nd	nd	nd
V68	Hexanoic acid, 2-methyl-	4536-23-6	16.74	nd	nd	nd
V69	Gamolenic acid	506-26-3	10.84	5.80	nd	nd
V70	2-Pentenoic acid, 4-methyl-	10321-71-8	10.22	nd	nd	nd
V71	Acrylic acid, 3,3-dimethyl-	541-47-9	5.96	nd	nd	nd
V72	Hexanoic acid, 5-methyl-	628-46-6	4.67	nd	1.19	nd
V73	Valeric acid, 4-methyl	646-07-1	5.26	nd	nd	nd
V74	Hexanoic acid	142-62-1	3.88	nd	nd	nd
V75	Acetic acid	64-19-7	nd	nd	1.32	nd
	**Esters**		**10.77**	**33.59**	**29.63**	**25.55**
V76	Hexanoic acid, ethyl ester	123-66-0	4.02	6.33	7.25	8.11
V77	Anthranilic acid, methyl ester	134-20-3	3.71	nd	7.00	7.87
V78	4-Decenoic acid, ethyl ester, (E)-	76649-16-6	nd	4.09	nd	4.03
V79	Palmitic acid, ethyl ester	628-97-7	nd	7.11	nd	nd
V80	Propanoic acid, 2-methyl-, 3-hydroxy-2,2,4-trimethylpentyl ester	77-68-9	nd	3.10	1.54	1.70
V81	1,2-Benzenedicarboxylic acid, dimethyl ester	131-11-3	nd	nd	6.23	nd
V82	Octanoic acid, ethyl ester	106-32-1	2.01	0.65	1.33	0.76
V83	4-Decenoic acid, (Z)-, ethyl ester	7367-84-2	nd	nd	4.26	nd
V84	2,2,4-Trimethyl-1,3-pentanediol diisobutyrate	6846-50-0	nd	4.16	nd	nd
V85	Acetic acid, phenethyl ester	103-45-7	nd	nd	0.84	3.08
V86	Decanoic acid, ethyl ester	110-38-3	nd	3.24	nd	nd

Table S1 (Continued 4). Main flavor substances detected in four high-temperature Daqu samples from the southern Sichuan–northern Guizhou region.

No.	Volatile compounds	CAS Number	Average concentration (ng/g dry weight)			
			**ZS**	**MS**	**SJ**	**ZJ**
V87	9,12-Octadecadienoic acid, (Z,Z)-, ethyl ester	544-35-4	nd	2.84	nd	nd
V88	Lauric acid, ethyl ester	106-33-2	nd	1.47	nd	nd
V89	3,4-Dimethoxybenzoic acid, methyl ester	2150-38-1	nd	nd	1.18	nd
V90	Pentanoic acid, 3-methyl-2-oxo-, methyl ester	3682-42-6	1.03	nd	nd	nd
V91	Nonylic acid, ethyl ester	123-29-5	nd	0.59	nd	nd
	**Alcohols**		**288.49**	**103.13**	**385.15**	**324.14**
V92	Phenethyl alcohol	60-12-8	83.10	13.68	168.02	207.56
V93	3-Octanol	589-98-0	23.68	4.38	75.92	11.51
V94	Fragranol	30346-21-5	36.32	nd	12.23	nd
V95	Benzyl alcohol	100-51-6	7.50	nd	12.93	20.91
V96	.beta.-Ethylphenethyl alcohol	2035-94-1	13.48	8.13	7.60	7.54
V97	Cyclobutaneethanol	30820-22-5	nd	12.30	nd	22.56
V98	2-Pentanol, [R,(-)]-	31087-44-2	28.91	3.94	nd	nd
V99	1-Butanol, 3-methyl-	123-51-3	0.68	4.85	9.01	11.86
V100	1-Octen-3-ol	3391-86-4	10.64	3.03	6.14	4.10
V101	1-Hexanol, 2-ethyl-	104-76-7	3.13	14.59	3.82	2.28
V102	1-Heptanol	111-70-6	5.89	6.34	5.96	5.56
V103	1-Octanol	111-87-5	8.20	2.83	7.12	4.28
V104	1-Hexanol	111-27-3	5.62	2.67	6.65	6.58
V105	Linalool	78-70-6	nd	nd	21.18	nd
V106	8-dodecen-1-ol, (Z)-	40642-40-8	12.46	5.42	nd	nd
V107	p-Menth-1-en-8-ol	98-55-5	nd	0.75	13.01	nd
V108	2-Nonen-1-ol, (E)-	31502-14-4	7.52	nd	4.92	nd

Table S1 (Continued 5). Main flavor substances detected in four high-temperature Daqu samples from the southern Sichuan–northern Guizhou region.

No.	Volatile compounds	CAS Number	Average concentration (ng/g dry weight)			
			ZS	MS	SJ	ZJ
V109	2-Heptanol, 5-methyl-	54630-50-1	12.40	nd	nd	nd
V110	2-Nonanol	628-99-9	2.61	2.58	3.34	3.49
V111	2-Octen-1-ol, (E)-	18409-17-1	3.34	nd	2.50	2.05
V112	1-Nonanol	143-08-8	nd	7.60	nd	nd
V113	Furfuryl alcohol	98-00-0	nd	nd	3.47	3.96
V114	2-Propanol, 3-phenyl-	698-87-3	5.22	nd	2.19	nd
V115	2,3-Butanediol, [R-(R*,R*)]-	24347-58-8	nd	3.29	nd	3.58
V116	2-Heptanol	543-49-7	nd	1.69	1.83	2.11
V117	1,10-Decanediol	112-47-0	nd	nd	5.14	nd
V118	2,3-Butandiol	513-85-9	nd	nd	4.25	nd
V119	Heneicosyl alcohol	15594-90-8	4.12	nd	nd	nd
V120	3-Hexanol	623-37-0	4.05	nd	nd	nd
V121	1-Propanol, 2-phenyl-	1123-85-9	0.74	1.12	1.02	1.04
V122	2-Hexanol	626-93-7	3.56	nd	nd	nd
V123	2-Methoxyphenethyl alcohol	7417-18-7	nd	0.60	0.74	1.33
V124	Cedrol	77-53-2	nd	nd	2.62	nd
V125	Oleyl alcohol	143-28-2	2.10	nd	nd	nd
V126	3-Nonen-1-ol	10340-23-5	nd	2.04	nd	nd
V127	1-Heptanol, 6-methyl-	1653-40-3	1.76	nd	nd	nd
V128	2-Pentanol	6032-29-7	nd	nd	1.61	nd
V129	1-Pentanol	71-41-0	nd	nd	0.60	0.98
V130	3-Pentanol	584-02-1	1.44	nd	nd	nd
V131	1-Propanol, 2-Methyl-	78-83-1	nd	nd	1.35	nd

Table S1 (Continued 6). Main flavor substances detected in four high-temperature Daqu samples from the southern Sichuan–northern Guizhou region.

No.	Volatile compounds	CAS Number	Average concentration (ng/g dry weight)			
			**ZS**	**MS**	**SJ**	**ZJ**
V132	Cyclohexylmethanol	100-49-2	nd	1.28	nd	nd
V133	4-Methoxyphenethyl alcohol	702-23-8	nd	nd	nd	0.86
	**Phenols**		**42.37**	**6.12**	**15.53**	**17.17**
V134	4-Vinylphenol, 2-methoxy-	7786-61-0	16.22	nd	6.22	6.60
V135	Ethylquinol	2349-70-4	19.38	nd	nd	nd
V136	1-Naphthol	90-15-3	nd	2.57	5.42	nd
V137	2,4-Di-tert-butylphenol	96-76-4	1.46	3.55	nd	2.85
V138	2-Naphthol	135-19-3	nd	nd	nd	7.72
V139	3-Ethylphenol	620-17-7	3.42	nd	nd	nd
V140	Hinokitiol	499-44-5	nd	nd	2.67	nd
V141	4-Cresol	106-44-5	1.90	nd	nd	nd
V142	3,5-Di-tert-butylphenol	1138-52-9	nd	nd	0.95	nd
V143	2-Cresol	95-48-7	nd	nd	0.27	nd
	**Ethers**		**10.80**	**23.04**	**9.65**	**15.44**
V144	Veratrole	91-16-7	10.80	7.15	8.06	12.76
V145	4-Methoxyanisole	150-78-7	nd	14.54	1.59	2.68
V146	Isopropylpropyl ether	627-08-7	nd	1.35	nd	nd
	**Others**		**14.81**	**11.21**	**12.78**	**21.13**
V147	Dimethyltrisulfide	3658-80-8	5.44	1.84	5.08	7.65
V148	Nonanolactone	104-61-0	3.76	0.58	5.49	5.41
V149	3-(1,1-Dimethylethyl)-1,2-dihydronaphthalene	55682-81-0	2.10	0.61	nd	3.03
V150	Benzothiazole	95-16-9	nd	4.25	nd	nd
V151	1-Butanamine, N-(2-furanylmethylene)-3-methyl-	52074-26-7	nd	nd	nd	3.53

Table S1 (Continued 7). Main flavor substances detected in four high-temperature Daqu samples from the southern Sichuan–northern Guizhou region.

No.	Volatile compounds	CAS Number	Average concentration (ng/g dry weight)			
			ZS	MS	SJ	ZJ
V152	Indole	120-72-9	1.82	1.18	nd	nd
V153	3-Methylbutyraldehyde oxime	626-90-4	1.69	nd	nd	nd
V154	Benzeneacetamide, α-hydroxy-N-methyl-	2019-72-9	nd	nd	1.56	nd
V155	4-Hydroxyquinazoline	491-36-1	nd	nd	0.65	0.77
V156	2-Anisidine	90-04-0	nd	1.28	nd	nd
V157	Anethole	104-46-1	nd	0.84	nd	nd
V158	3-Phenylthiophene	2404-87-7	nd	nd	nd	0.75

Table S2. Statistics of metagenomic sequencing and bioinformatics analysis.

Parameter	ZS	MX	SJ	ZJ
**Sequencing**				
Raw base (bp)	19071515628	16691106630	20109984908	21532002342
Raw Q20 (%)	98	97	98	98
Raw Q30 (%)	96	94	95	95
Clean base(bp)	18737722769	16061337782	19825829362	21268700590
clean Q20(%)	98.79	98.57	98.64	98.66
clean Q30(%)	96.19	95.64	95.79	95.84
clean GC(%)	39.98	43.82	47.04	47.29
high-quality reads(%)	99.10	96.69	99.10	99.37
**Assembly**				
contigs number	534798	381327	251623	290512
N50 length(bp)	1656	3406	34343	10680
N90 length(bp)	1038	1193	1332	1392
Gene Prediction				
ORFs number	444720	529064	502978	534211
unique number	981430	959176	1262418	1120096

Table S3. Results of LDA discrimination analysis at the species level of microbial community.

Species name	Group	Mean	LDA value	P value
**Fun.**				
Aspergillus chevalieri	ZS	5.18	4.86	0.016
Lichtheimia ramosa	ZS	5.08	4.63	0.016
Lichtheimia ornata	ZS	4.82	4.46	0.016
Aspergillus cristatus	ZS	4.53	4.12	0.019
Lichtheimia corymbifera	ZS	4.46	4.08	0.016
Monascus purpureus	MX	5.32	4.98	0.016
Paecilomyces variotii	SJ	5.23	4.24	0.031
Rasamsonia emersonii	ZJ	5.28	4.79	0.024
**Bac.**				
Lentibacillus daqui	ZS	5.48	5.14	0.016
Bacilli bacterium VT-13-104	MX	4.40	4.10	0.016
Oceanobacillus caeni	MX	4.69	4.40	0.016
unclassified f_Bacillaceae	MX	4.87	4.57	0.016
Saccharopolyspora rectivirgula	SJ	4.76	4.41	0.016
unclassified g_Thermoactinomyces	SJ	4.88	4.55	0.022
Weissella confusa	SJ	4.38	4.09	0.016
Desmospora sp. 8437	ZJ	5.05	4.55	0.016
Kroppenstedtia eburnea	ZJ	5.38	4.89	0.016
Kroppenstedtia guangzhouensis	ZJ	4.73	4.27	0.024
Limosilactobacillus pontis	ZJ	4.32	4.03	0.019

Table S4. Description of each COG category.

Category	Functional description
A	RNA processing and modification
B	Chromatin structure and dynamics
C	Energy production and conversion
D	Cell cycle control, cell division, chromosome partitioning
E	Amino acid transport and metabolism
F	Nucleotide transport and metabolism
G	Carbohydrate transport and metabolism
H	Coenzyme transport and metabolism
I	Lipid transport and metabolism
J	Translation, ribosomal structure and biogenesis
K	Transcription
L	Replication, recombination and repair
M	Cell wall/membrane/envelope biogenesis
N	Cell motility
O	Posttranslational modification, protein turnover, chaperones
P	Inorganic ion transport and metabolism
Q	Inorganic ion transport and metabolism
S	Unknow function
R	General function prediction only
T	Signal transduction mechanisms
U	Intracellular trafficking, secretion, and vesicular transport
V	Defense mechanisms
W	Extracellular structure
Z	Cytoskeleton

Table S5. Description of each KEGG code.

Code	Functional description
a	Global and overview maps
b	Glycan biosynthesis and metabolism
c	Carbohydrate metabolism
d	Amino acid metabolism
e	Metabolism of cofactors and vitamins
f	Lipid metabolism
g	Energy metabolism
h	Xenobiotics biodegradation and metabolism
i	Biosynthesis of other secondary metabolites
j	Nucleotide metabolism
k	Metabolism of other amino acids
l	Metabolism of terpenoids and polyketides

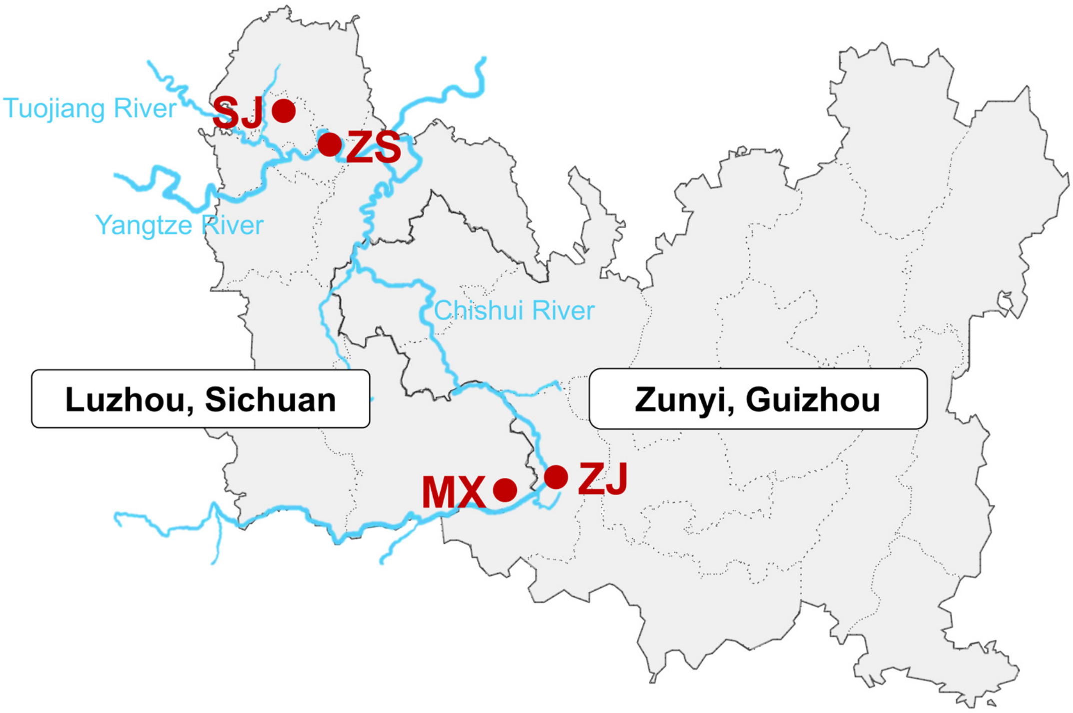

Figure S1. Geographical distribution of high-temperature Daqu in the southern Sichuan–northern Guizhou Region.

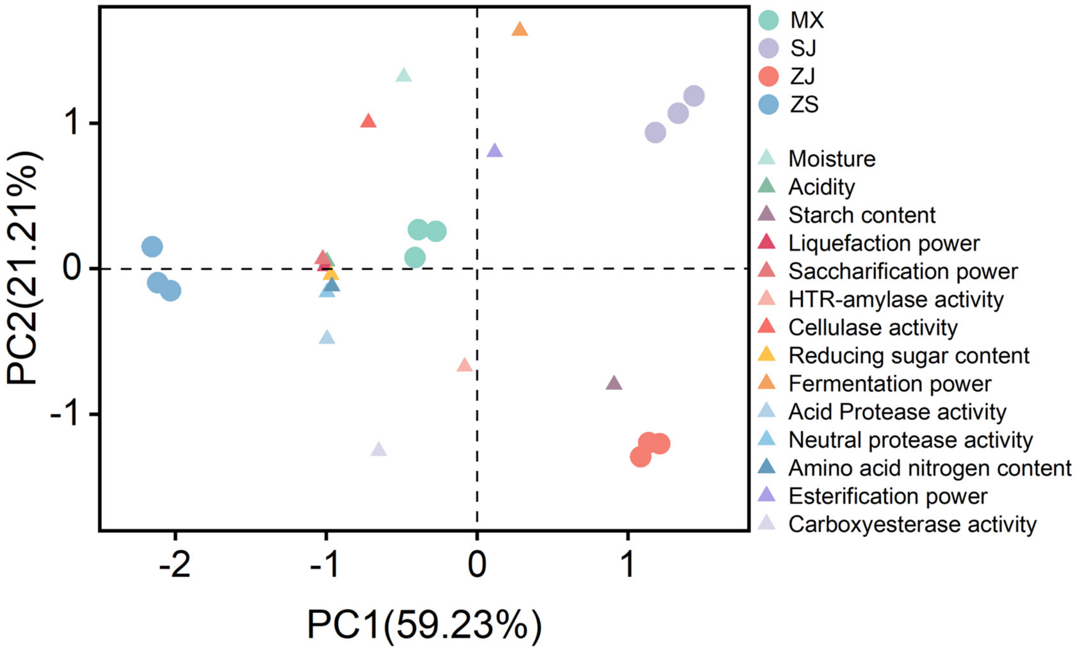

Figure S2. Score plot and loading plot of principal component analysis (PCA) reveal the varied physicochemical profiles among the four high-temperature Daqu samples.

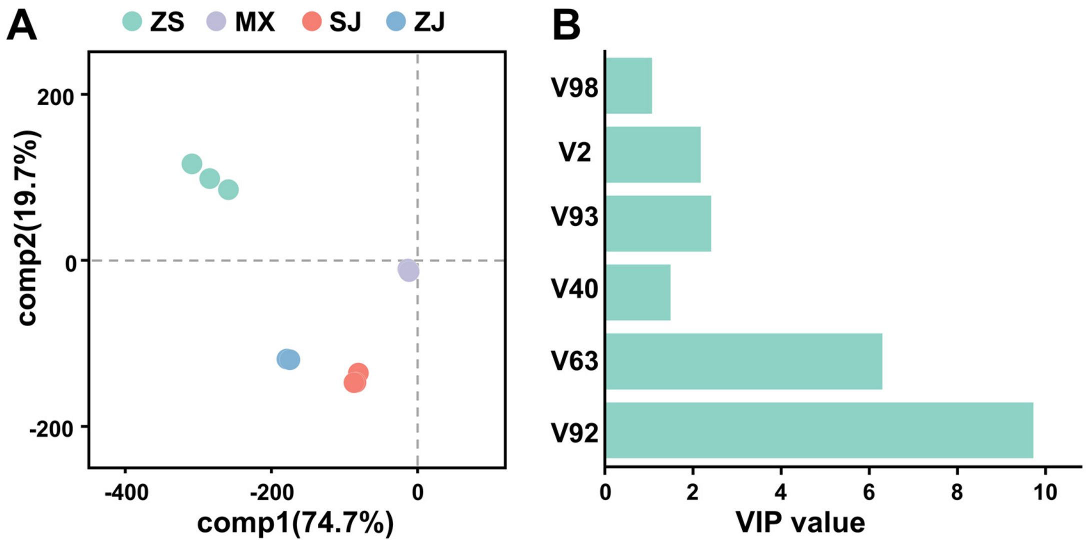

Figure S3. Ordination plot (A)and VIP plot (B) of partial least-squares discriminant analysis (PLS-DA) reveal the volatile compounds with significant differences in the four high-temperature Daqu samples.

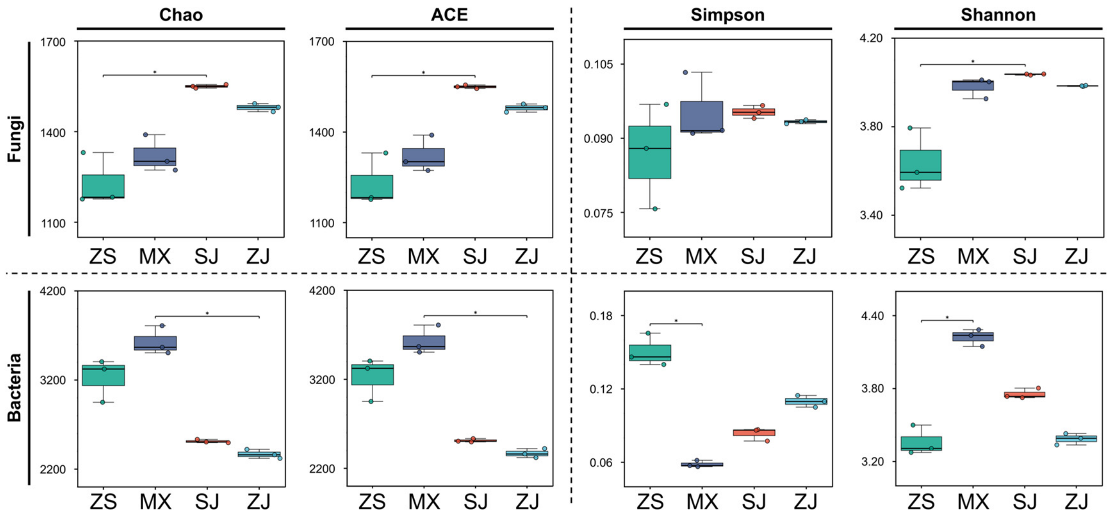

Figure S4. Differences in α-diversity indices of microbial communities among the four high-temperature Daqu samples.

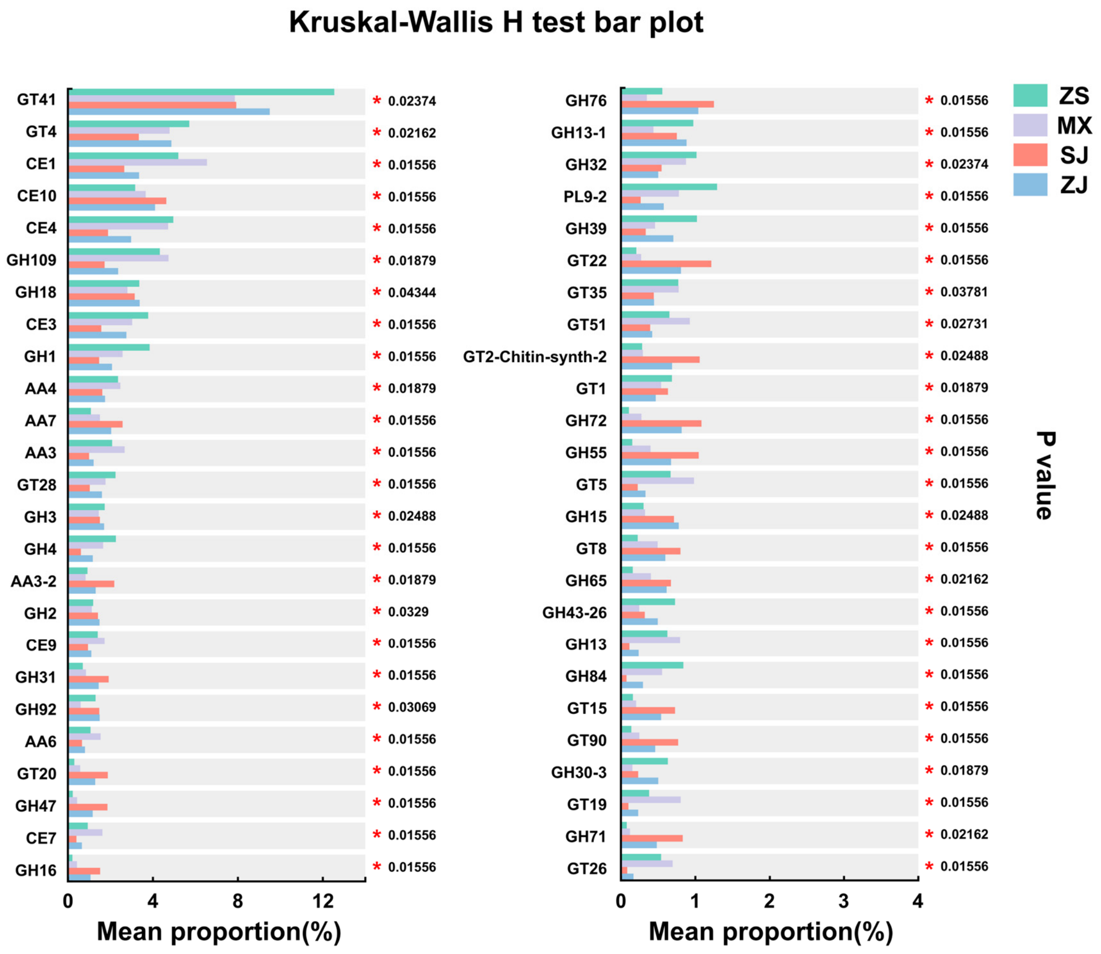

Figure S5. Kruskal–Wallis H test bar plot of the top 50 differential enzyme families in terms of abundance among the four high-temperature Daqu samples.
